# Molecular characterization of ESBL-producing *Escherichia coli* from UTIs and the antimicrobial activity of biosynthesized selenium nanocomposites

**DOI:** 10.3389/fcimb.2026.1774867

**Published:** 2026-04-07

**Authors:** Heba A. Ahmed, Abdul-Raouf Al-Mohammadi, Noha A. Anwar, Eman Y. T. Elariny, Nashwa El-Gazzar

**Affiliations:** 1 Department of Zoonoses, Faculty of Veterinary Medicine, Zagazig University, Zagazig, Egypt; 2 Department of Science, King Khalid Military Academy, Riyadh, Saudi Arabia; 3 Department of Botany and Microbiology, Faculty of Science, Zagazig University, Zagazig, Egypt

**Keywords:** ESBL-producing *E. coli*, phylotyping, selenium nanocomposites, UTIs, virulotyping

## Abstract

The spread of extended-spectrum β-lactamase (ESBL)-producing *Escherichia coli* causing urinary tract infections (UTIs) is a growing public health concern. The objectives of this study were to determine the pathotypes, virulotypes, genotypes, and antimicrobial resistance patterns of ESBL-producing *E. coli* isolated from patients with UTI in Egypt and evaluate the bactericidal efficacy of selenium nanoparticles (SeNPs) and selenium nanocomposites (SeNCs) against multidrug-resistant (MDR) isolates. We characterized 20 ESBL-producing *E. coli* from 41 clinical isolates recovered from urine and stool of hospitalized patients with UTI, by phylogrouping, virulence genes profiling, repetitive extragenic palindromic elements–polymerase chain reaction (REP-PCR) genotyping, and antibiotic susceptibility testing. ESBL phenotypes were confirmed by standard disc diffusion tests; resistance to ampicillin and cefixime was the most common. Virulence profiling identified the *fim*H gene as the most frequent identified gene. High strain diversity was observed by REP-PCR genotyping. To generate SeNCs that act as inhibitory agents against pathogenic microbes, this study combined SeNPs with cefoperazone (CEP) for SeNCs formation. *Aspergillus fumigatus* was used for the biosynthesis of SeNPs. SeNPs and SeNCs have potential antibacterial activities against ESBL-producing *E. coli* with a minimum inhibitory concentration (MIC) of 20 and 10 μg/mL, respectively. In addition, transmission electron microscopy (TEM) images of *E. coli* with SeNCs exhibited wrinkled external surfaces, asymmetric cell deformations, and cell depressions. In conclusion, virulent MDR ESBL-producing *E. coli* isolates were identified in samples from patients with UTI in Egypt, posing significant public health threats. Regular monitoring of the prevalence and antimicrobial resistance profile of ESBL-producing *E. coli* is crucial. SeNCs exhibited significantly more antibacterial activities than SeNPs and CEP.

## Introduction

Urinary tract infections (UTIs) are among the most common bacterial infectious diseases affecting over 150 million individuals, leading to high morbidity and healthcare burden (Niranjan and Malini, 2014; Mlugu et al., 2023). Members of the *Enterobacterales* family are the principle causative agents, with uropathogenic *Escherichia coli* (UPEC) responsible for the majority of community and nosocomial acquired infections (Ejrnaes, 2011; Abongomera et al., 2021). UPEC colonize the urinary tract and may cause cystitis and pyelonephritis, which can progress to urosepsis (Belanger et al., 2011).

The gastrointestinal tract is widely recognized as the primary source of UPEC, and intestinal colonization usually precedes ascending UTI (Shreiner et al., 2015; Mestrovic et al., 2020). Accordingly, comparative analysis of *E. coli* isolates from stool and urine samples may provide insights into endogenous transmission, shared virulence determinants, and resistance patterns.

The global rise of antimicrobial resistance (AMR) is a public health concern and significantly compromised the treatment of UTIs (Salam et al., 2023). β-lactam resistance among uropathogens is emerging due to the widespread dissemination of extended-spectrum β-lactamases (ESBLs), which hydrolyze third-generation cephalosporines and limit their therapeutic effect (Abongomera et al., 2021). The emergence/reemergence of multidrug-resistant (MDR) strains has further complicated treatment strategies, highlighting the need for continuous surveillance of resistance patterns and molecular determinants (Antimicrobial Resistance Collaborators, 2022; Razaq et al., 2024).

Broad-spectrum antibiotics such as fluoroquinolones, penicillin, and cephalosporins are commonly used for the treatment of UTIs (Husna et al., 2023). However, the acquisition of ESBLs is the primary cause of emerging resistance to β-lactam antibiotics such as ampicillin, cefotaxime, ceftazidime, and aztreonam (Mohamed et al., 2020; Husna et al., 2023). ESBL genes are plasmid mediated, facilitating the horizontal transfer of these genes to other species and genera, thus contributing to the dissemination of MDR strains (Azab et al., 2021; Husna et al., 2023). These plasmids often harbor coexisting genes encoding resistance to non-β-lactam antimicrobials such as quinolones and aminoglycosides, further limiting therapeutic options (Ugbo et al., 2020).

*E. coli* pathogenicity is associated with diverse virulence determinants, including adhesions (e.g., fimbrial H gene, *fim*H), toxins (e.g., hemolysin, *hly*), and Shiga toxins (*stx*1 and *stx*2) (Ahmed et al., 2023). In addition, based on the presence or absence of *Chu*A, *yja*A, *TspE4.C2*, and *arpA* genes, *E. coli* are categorized into eight phylotypes, among them B2 and D are mainly associated with extraintestinal diseases (Gordon et al., 2008; Allen et al., 2010; Clermont et al., 2014).

Molecular typing methods such as repetitive extragenic palindromic elements–polymerase chain reaction (REP-PCR) further allow assessment of genomic diversity, clonal relationships, and the source of infection among isolates (Versalovic et al., 1991; Olive and Bean, 1999; Healy et al., 2005). Characterization of virulence determinants, phylotyping, and genotyping provide insights into the epidemiology and dissemination of ESBL-producing strains. However, there are limited data regarding the combined molecular characterization, virulotyping, and genotyping among ESBL-producing *E. coli* from UTIs.

The rising frequencies of MDR *E. coli* in UTIs, driven in part by inappropriate antibiotics use, highlight the need for alternative strategies (Clermont et al., 2014). Nanotechnology-based approaches have attracted increasing interest as alternative tools to enhance antibacterial efficacy. Selenium nanoparticles (SeNPs) and selenium nanocomposites (SeNCs) were selected in this study due to their reported broad-spectrum interaction with the cell surface of Gram-negative bacteria, affecting bacterial permeability and thus causing damage and structural changes (Mikhailova, 2023). Furthermore, SeNPs disrupt bacterial membranes, leading to cell death by compromising cell structure and function (Hegerova et al., 2015). In addition, nanocomposites incorporated with cefoperazone (CEP) act as drug delivery systems and exhibit enhanced antibacterial effects against diverse MDR pathogens (Sitohy et al., 2021). However, the effectiveness of such nanomaterials against ESBL-producing *E. coli* isolates remains insufficiently investigated.

Given the limited data on the molecular epidemiology of ESBL-producing *E. coli* from patients with UTI and the need for alternative therapeutic strategies against MDR isolates, this study aimed to investigate the molecular characteristics and AMR profile of ESBL-producing *E. coli* isolated from urine and stool samples of patients with UTIs. Moreover, we aimed to evaluate the *in vitro* antibacterial activity of SeNPs and SeNCs against these isolates.

## Materials and methods

### Isolates

Forty-one *E. coli* isolates were obtained from a previously conducted study that involved urine (*n* = 31) and stool (*n* = 10) isolates collected from 441 hospitalized patients with UTI at Sednawy, Al-Ahrar, and The University Hospitals, Zagazig City, Sharkia Governorate, Egypt, between September and December 2019. The current study involved laboratory analysis of these previously isolated and anonymized isolates only. Ethical approval for the original sample collection was obtained from the Research Ethical Committee, Faculty of Medicine, Zagazig University (IRB approval number ZU-IRB#1005) and in accordance with the principles outlined in the Declaration of Helsinki (https://www.wma.net/what-we-do/medical-ethics/declaration-of-helsinki/). No additional recruitment of patients was performed for the present study.

### Antimicrobial resistance profile

The susceptibility of 41 *E. coli* isolates against 13 antibacterial agents was examined according to the guidelines of the Clinical Laboratory Standards Institute (CLSI, 2020). The agents used were provided by Bioanalyze Company (Turkey), including penicillins (Ampicillin (AM), 20 μg), fluoroquinolones [ofloxacin (OFX), 5 μg; levofloxacin (LEV), 5 μg; and ciprofloxacin (CIP), 5 μg], quinolones [nalidixic acid (NA), 30 μg], aminoglycopeptides [gentamicin (CN), 10 μg; kanamycin (K), 30 μg; and amikacin (AK), 30 μg], cephalosporins [Cefoperazone (CEP), 30 μg and cefixime (CFM), 10 μg], sulfonamides [trimethoprim/sulfamethoxazole (SXT), 25 μg], and carbapenems [imipenem (IPM), 10 μg and meropenem (MEM), 10 μg]. *E. coli* ATCC 25922 was used as the quality control organism.

The multiple antibiotic resistance (MAR) index was calculated as the ratio of the number of antibiotics to which *E. coli* isolates displayed resistance to the number of drugs to which *E. coli* isolates were exposed (Krumperman, 1983). MDR isolates are defined as isolates resistant to at least one agent from three or more antibiotic classes, while extensively drug resistant (XDR) isolates are defined as isolates resistant to at least one agent in all but two or fewer antimicrobial categories (Magiorakos et al., 2012).

### Screening of ESBL-producing *E. coli* isolates

Isolates showing a zone of inhibition of ≤22 mm for ceftazidime, ≤27 mm for cefatoxime, and ≤25 mm for ceftriaxone were subjected to ESBL confirmatory tests, namely, the Double Disc Synergy Test (DDST) and the Phenotypic Confirmatory Disc Diffusion Test (PCDDT) (Shukla et al., 2004; Kolhapure et al., 2015).

### Amplification of ESBL production-associated genes

Bacterial isolates were cultured overnight on nutrient agar at 37°C prior to DNA extraction. The DNA from the isolates was extracted using the QIAamp DNA Mini kit (QIAGEN, GmbH, Hilden, Germany) according to the manufacturer’s guidelines. *bla*IMP, *bla*VIM, NDM-1 (Xia et al., 2012), *bla*TEM, and *bla*SHV (Colom et al., 2003) were amplified by conventional polymerase chain reaction (PCR). The primers were synthesized by Midland Certified Reagent Company-Oligos (USA) and the sequences are listed in **Supplementary Table 1**. The reaction was performed in 25 μL volume containing 12.5 μL of readymade power Emerald Amp GTPCR Master mix (Takara), 20 pmol of each primer (1 μL, each), 6 μL of purified DNA, and 4.5 μL of PCR grade water. A negative control (reaction mixture without adding DNA) and a positive control (provided by the Reference Laboratory for Veterinary Quality Control on Poultry Production, Animal Health Research Institute, Dokki, Giza) were included in each run. The cycling conditions included initial denaturation at 94°C for 5 min, followed by 35 cycles of denaturation at 94°C for 30 s, annealing at the specified temperature for each primer (**Supplementary Table 1**), extension at 72°C for 40 s, and a final extension at 72°C for 10 min.

### ESBL-producing *E. coli* phylotyping, virulotyping, and genotyping

ESBL-producing *E. coli* were phylotyped using primers for the amplification of *chu*A, *yja*A, and *tspE4C2* genes according to the phylotype classification scheme previously described (Jeong et al., 2012).

Virulotyping was also performed using specific primers for amplification of the virulence-associated genes including *fimH* (Ghanbarpour and Salehi, 2010), *hly* (Piva et al., 2003), *stx2* (Dipineto et al., 2006), *eaeA* (Bisi-Johnson et al., 2011), and *stx2f* (Schmidt et al., 2000).

Genotyping was performed on extracted DNA through fingerprinting PCR using REP-primers synthesized by Metabion (Germany). The primers’ sequences are 5′-IIIICGICGICATCIGGC-3′ and 5′-ICGICTTATCIGGCCTAC-3′ (Mohapatra et al., 2007). The primers were used in a 25-µL reaction mixture containing 12.5 µL of EmeraldAmp Max PCR Master Mix (Takara, Japan), 1 µL of each primer at a concentration of 20 pmol, 4.5 µL of water, and 6 µL of DNA template. The PCR reaction was conducted using an Applied Biosystem 2720 thermal cycler. The REP-PCR fingerprinting data were transformed into a binary code based on the presence or absence of each band, and the discriminatory power of the reaction was measured using the Simpson’s index of diversity (*D*). A *D* value of more than 0.9 indicated good differentiation (Hunter, 1990).

### Effect of SeNPs and SeNCs on *E. coli* isolates

#### Biosynthesis of SeNPs and synthesis of SeNCs

SeNPs were biosynthesized following a previous method (El-Gazzar and Ismail, 2020), by utilizing a salt of selenium sulfate metal at 1 mM (Nanotech Company, Dream land, Giza, Egypt) with *Aspergillus fumigatus*, then incubated at 28°C until the biosynthesis of SeNPs.

*A. fumigatus* was incubated at 30°C with shaking at 150 rpm for 5–7 days. The fungal culture was then filtered, and the fungal mats were collected and re-inoculated in deionized water, followed by incubation under the same condition. After incubation, the culture was filtered again and the obtained filtrate was treated with selenium sulfate (1 mM) at 25°C for 24 h, resulting in SeNP formation as indicated by a visible color change. Subsequently, nanoparticles were characterized. Purification was performed by centrifugation at 12,000 × *g* for 15 min followed by triple washing with sterile deionized water to remove unreacted precursors and residual biomolecules. The purified SeNPs were stored in sterile amber vials at 4°C and were protected from light (El-Gazzar and Ismail, 2020).

The fungal isolate that produced SeNPs was identified at Assiut University’s Molecular Unit and then stored at the Molecular Culture Collection (AUMC). Before being sent to SolGent Company in Daejeon, South Korea, for PCR and 18S rRNA gene sequencing, the DNA was stored in 1.5-mL autoclaved Eppendorf tubes. The reaction mixture was supplemented with ITS1 (forward) and ITS4 (reverse) primers, with the respective sequences ITS1 (5′-TCC GTA GGT GAA CCT GCG G-3′) and ITS4 (5′-TCC TCC GCT TAT TGA TAT GC-3′). The purified PCR material was sequenced using the same primers after ddNTPs were added to the reaction mixture (White et al., 1990). The National Centre for Biotechnology Information (NCBI) website’s Basic Local Alignment Search Tool (BLAST) was used for thorough examination of the gathered sequences. MegAlign (DNA Star) software version 5.05 was used to perform phylogenetic analysis on the sequences (El-Gazzar et al., 2025).

SeNCs were synthesized by the sonication method, where 1 g of SeNP dispersion in distilled H_2_O (200 mL) was subjected to 1 mL of CEP under a sono-condition of 0.5 cycles and 50% amplitude for 2 h until a homogeneous composite was produced (Ismail et al., 2020).

### Characterization of both SeNPs and SeNCs

SeNPs were monitored using an ultraviolet–visible (UV–Vis) spectrophotometer (double-beam UV–Vis spectrophotometer); UV–Vis spectroscopy measures the absorption of UV and visible light by a sample as a function of wavelength. SeNPs exhibit a characteristic surface plasmon resonance (SPR) absorption band in the 265- to 350-nm regions, arising from collective oscillation of conduction electrons. CEP, a third-generation cephalosporin antibiotic, shows strong absorption at approximately 254 nm (Lashani et al., 2024). The colloidal SeNP suspension was diluted to a concentration of 100 μg/mL using deionized water. The solution was sonicated for 5 min to ensure uniform dispersion prior to measurement. Free cefoperazone sodium (Cefozon, EIPICO, 2 g) was dissolved in deionized water to achieve a final concentration of 50 µg/mL. The solution was freshly prepared and protected from light. The nanocomposite suspension was diluted to 100 μg/mL with deionized water and sonicated for 10 min to achieve complete dispersion. The solution was allowed to equilibrate at room temperature for 5 min before measurement.

The spectrophotometer was warmed up for 30 min prior to measurements to ensure lamp stability. Baseline correction was performed using deionized water in matched quartz cuvettes. Each sample was scanned in triplicate across the wavelength range of 200–800 nm. Spectra were recorded at a scan speed of 400 nm/min with a 1.0-nm sampling interval. The absorbance values were recorded and averaged for the three replicates.

SeNPs and SeNCs were subjected to a dynamic light scattering (DLS) system to determine the particles’ diameters (El-Gazzar and Ismail, 2020).

Zeta potential measurements were conducted using a Malvern Zetasizer Nano ZS instrument (Malvern Panalytical, UK) with factory-calibrated DTS1235 disposable folded capillary cells. Colloidal suspensions of SeNPs and SeNPs–CEP nanocomposite were diluted to an appropriate concentration (~0.1 mg/mL) using deionized water to achieve an attenuator position between 6 and 8. Samples were sonicated for 5 min in an ultrasonic bath prior to measurement. Approximately 1 mL of each sample was injected into a DTS1070 folded capillary cell using a 1-mL syringe, avoiding air bubbles. The cell was allowed to equilibrate at 25°C for 120 s before measurement. Zeta potential determination employed electrophoretic light scattering with automatic voltage optimization and consisted of 15 measurement runs with inter-run equilibration periods. Results represent the mean ± standard deviation of three independent measurements. Instrument performance was verified daily using certified zeta potential transfer standards (Malvern DTS1235, nominal ζ = −42 ± 4.2 mV, acceptance criterion: measured value within 5% of nominal) (El-Gazzar and Ismail, 2020).

Furthermore, transmission electron microscopy (TEM) analysis was performed on SeNPs and SeNCs. In double-deionized water, SeNPs or SeNCs were added, and the mixture was dispersed at 50 kHz, with an amplitude of 85% and a cycle length of 0.65 at 50 min (UP400S, Hielscher, Germany). The sample was then aliquoted (5 µm) and applied on a grid of copper for TEM studies (Sitohy et al., 2021).

SeNPs and SeNCs were characterized by Fourier transform infrared spectroscopy (FTIR) (Thermo Nico let model 6700 spectrum) (El-Gazzar and Ismail, 2020). The colloidal SeNPs suspension was centrifuged at 12,000 rpm for 15 min. The pellet was washed three times with deionized water and dried at 60°C overnight in a vacuum oven. The dried powder was placed directly on the ATR crystal. CEP sodium powder (Cefozon, EIPICO, 2 g) was used directly without further preparation. A small amount of powder was placed on the ATR crystal and pressed uniformly. The SeNPs–CEP nanocomposite was centrifuged at 12,000 rpm for 15 min, washed three times with deionized water to remove unbound drug, and dried at 60°C overnight under vacuum. The dried nanocomposite powder was analyzed directly on the ATR crystal.

Background spectrum was collected using a clean ATR crystal with 32 scans. The sample was placed on the ATR crystal with uniform pressure applied via the pressure clamp. Each sample was scanned 32 times and averaged for improved signal-to-noise ratio. The ATR crystal was cleaned with ethanol and acetone between samples. Measurements were performed in triplicate for each sample. Atmospheric CO_2_ and H_2_O corrections were applied automatically.

XRD was conducted using a Bruker, D8 discover set (Billerica, MA, USA) to establish the character of the colloid and assess the homogeneity and purity of SeNPs, antibiotic, and SeNCs (El-Gazzar et al., 2025).

SeNPs and SeNCs were assessed via energy dispersive-x-ray spectroscopy (EDS). A drop of the nanoparticle suspension was deposited on a clean silicon wafer and dried at room temperature overnight. The sample was sputter-coated with a thin gold layer (~5 nm) to prevent charging artifacts (Mikhailova, 2023).

### Antibacterial efficiency of SeNPs and SeNCs

The antibacterial activity of SeNPs and SeNCs was assessed on *E. coli* isolates using the agar well diffusion method. The procedure involved inoculating 0.1 mL of a 24-h-old bacterial solution separately into 25 mL of melted Muller Hinton agar. The liquid had been put into a Petri dish and allowed to harden for 30 min at room temperature. One hundred microliters of the SeNPs and SeNCs supernatant was added to each well. Positive control wells contained broth medium supplemented with antibiotic (10 mg/mL), while negative control wells contained broth medium only. The inhibition diameters were determined after the plates had been incubated for 24 h at 37°C (Abou Elez et al., 2021).

### Minimum inhibitory concentration analysis

The MIC values were estimated by broth dilution procedures versus *E. coli* isolates according to CLSI guidelines (CLSI, 2018), following EUCAST recommendations for standardized MIC reporting (EUCAST, 2023). SeNPs and SeNCs were separately prepared at various dilutions of 10 to 100 μg/mL. Fresh microbial cultures (100 µL) were adjusted to a final inoculum concentration of approximately 5 × 10^5^ CFU/mL and then inoculated into tubes treated with SeNPs and SeNCs dilutions and incubated at 37°C for 18–24 h. Growth controls (medium with inoculum) were included. Every dilution was evaluated in triplicate in a separate experiment. Microbial growth was then read visually. The lowest concentration of nanoparticles that inhibited visible microbial growth was recorded as the MIC values (µg/mL).

### Transmission electron microscopy analysis

SeNPs and SeNCs were added to *E. coli* culture cultivated for 24 h on nutrient broth media. Following a 10-min centrifugation at 4,000 rpm, the bacterial cells were fixed with 3% glutaraldehyde, washed with phosphate buffer, and then fixed once more in a potassium permanganate solution for 5 min at room temperature. After 15 min of dehydration in an ethanol series ranging from 10% to 90%, the samples were subjected to 30 min of absolute ethanol. Epoxy resin and acetone were subjected to penetrate samples. On copper grids, ultrathin slices were cut out, double-stained with uranyl acetate and lead citrate, and then examined with a transmission electron microscope (JEOL JEM-1010, Tokyo, Japan). As a control, *E. coli* devoid of SeNPs and SeNCs was employed (Abou Elez et al., 2023).

### Statistical analysis

The data were analyzed using SPSS version 26 (IBM Corp, Armonk, NY, USA). Differences in the prevalence of *E. coli* isolates across sources, AMR patterns, and the distribution of investigated genes among isolates from different origins were assessed using Fisher’s exact test, with the chi-square test applied when its assumptions were satisfied. *p*-values less than 0.05 were considered statistically significant. Before correlation analyses, variables were tested for normality using a Q–Q plot. The variables—*bla*VIM, *bla*IMP, *bla*TEM, *bla*SHV, and *fim*H genes—were excluded from the analyses as they were identical to all isolates under study. Pairwise associations among the remaining variables were evaluated using Pearson’s correlation coefficient (*r*). To reduce the risk of Type I error due to multiple comparisons, *p*-values were adjusted using the Benjamini–Hochberg false discovery rate (FDR) correction. Correlation analyses and visualization were performed in R software version 4.3.3 (https://www.r-project.org/) using the Hmisc package (Harrell, 2003). Stacked bar plots, where sub-columns are part of the total column, were used for visualization of resistance to various antimicrobial agents and classes, distribution of resistance, and virulence genes. All graphs were generated by R software version 4.3.3 (R: The R Project for Statistical Computing) using ggplot (Wickham et al., 2007), pheatmap (Kolde, 2010), and factoextra (Kassambara and Mundt, 2016).

The data of SeNPs and Se-antibiotic-NCs effects were checked for normal distribution using the Shapiro–Wilk test. Results are presented as mean ± standard deviation (SD). Statistical significance was assessed using one-way analysis of variance (ANOVA) with *post-hoc* pairwise comparisons adjusted by the Tukey test to determine differences between different groups. Repeated-measures ANOVA was used to compare conditions and intervals within each strain, with pairwise comparisons using *post-hoc* Bonferroni adjustment.

## Results

**Figure 1:**
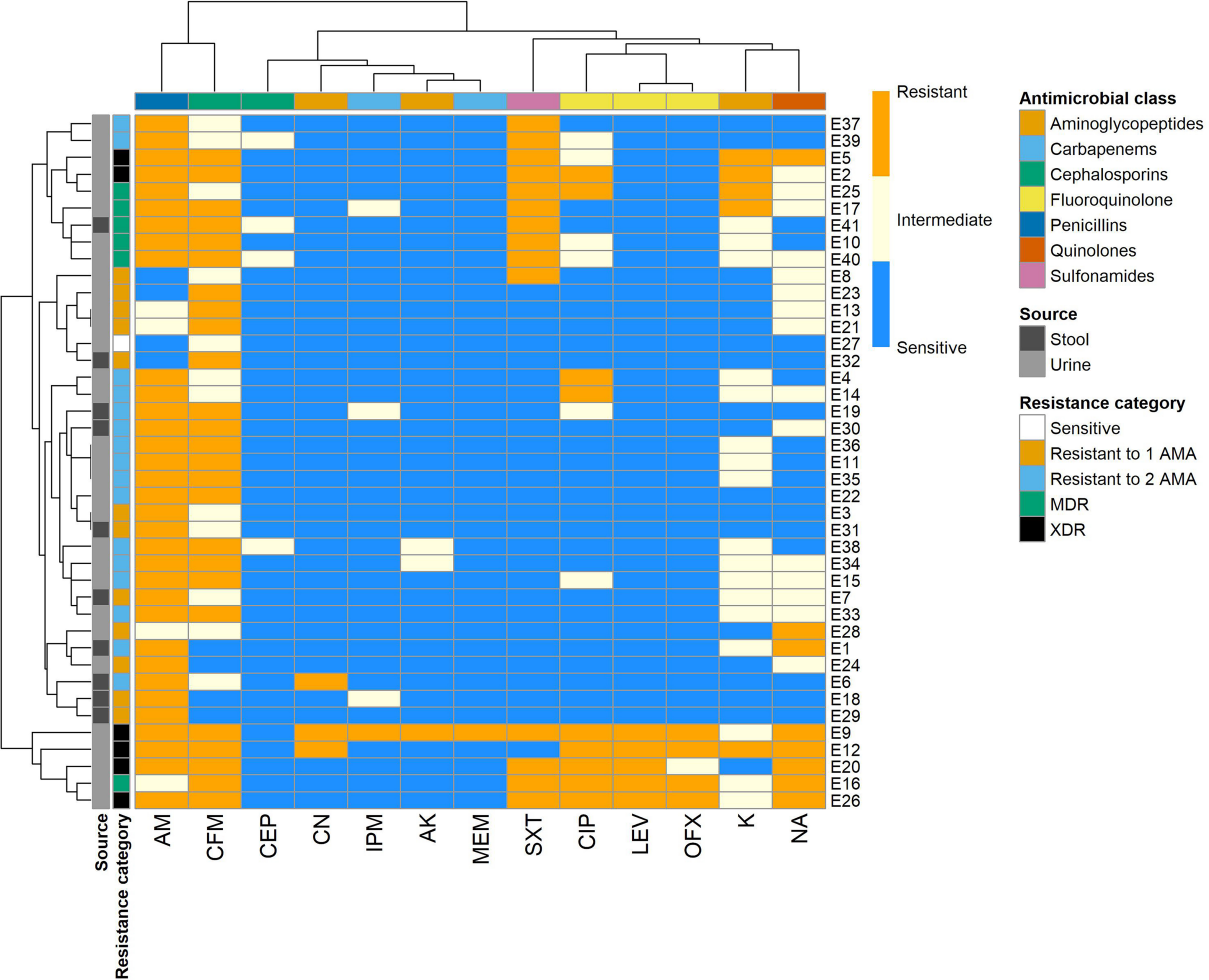
Hierarchical clustering heatmap showing the overall distribution of the investigated *Escherichia coli* isolates based on the phenotypic antimicrobial resistance pattern. Different sample sources, resistance categories, isolate pathotypes, isolate serotypes, and antimicrobial classes are color-coded on the right of the heatmap. ETEC, Enterotoxigenic *E. coli*; EPEC, Enteropathogenic *E. coli*; EIEC, Enteroinvasive *E. coli*; EHEC, Enterohemorrhagic *E. coli*; MDR, multidrug-resistant; XDR, extensive drug-resistant; AM, Ampicillin; K, Kanamycin; CN, Gentamicin; AK, Amikacin; CFM, Cefixime; CEP, Cefoperazone; SXT, Trimethoprim/sulfamethoxazole; MEM, Meropenem; IPM, Imipenem; CIP, Ciprofloxacin; LEV, Levofloxacin; OFX, Ofloxacin; NA, Nalidixic acid.

**Figure 2:**
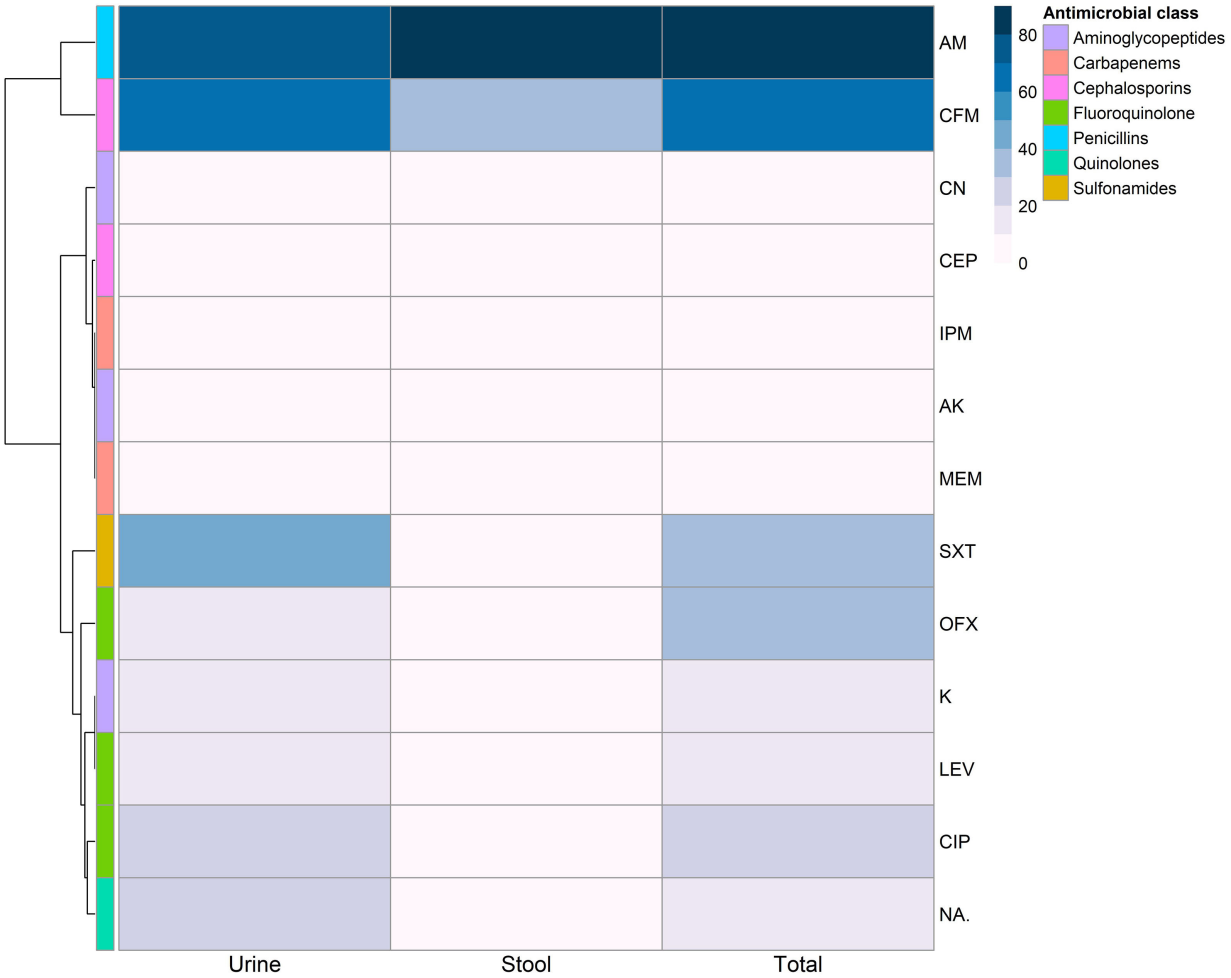
Antimicrobial resistance patterns of *Escherichia coli* isolates isolated from urine and stool samples. AM, Ampicillin; S, Streptomycin; K, Kanamycin; CN, Gentamicin; AK, Amikacin; CFM, Cefixime; CEP, Cefoperazone; SXT, Trimethoprim/sulfamethoxazole; MEM, Meropenem; IPM, Imipenem; CIP, Ciprofloxacin; LEV, Levofloxacin; OFX, Ofloxacin; NA, Nalidixic acid.

The antibiotic susceptibility of 41 *E. coli* isolates was tested against 13 antibiotics belonging to seven antimicrobial classes using the disc diffusion method (**Figures 1**, **2**). The most common resistance was observed against AM (33/41, 80.49%) and CFM (25/41, 60.98%), while a high level of sensitivity was observed for MEM (40/41, 97.56%), followed by CN and AK (38/41, 92.68%, each).

Out of the examined 31 *E. coli* isolates from urine, 24 (77.4%) were resistant to AM, while 21 (67.7%) were resistant to CFM. The isolates from stool also showed resistance to AM and CFM with respective percentages of 90% (9 out of 10 isolates) and 40% (4 out of 10 isolates). There was no statistically significant difference in the AMR patterns of the tested *E. coli* isolates from urine and stool samples (*p* > 0.05).

**Figure 3:**
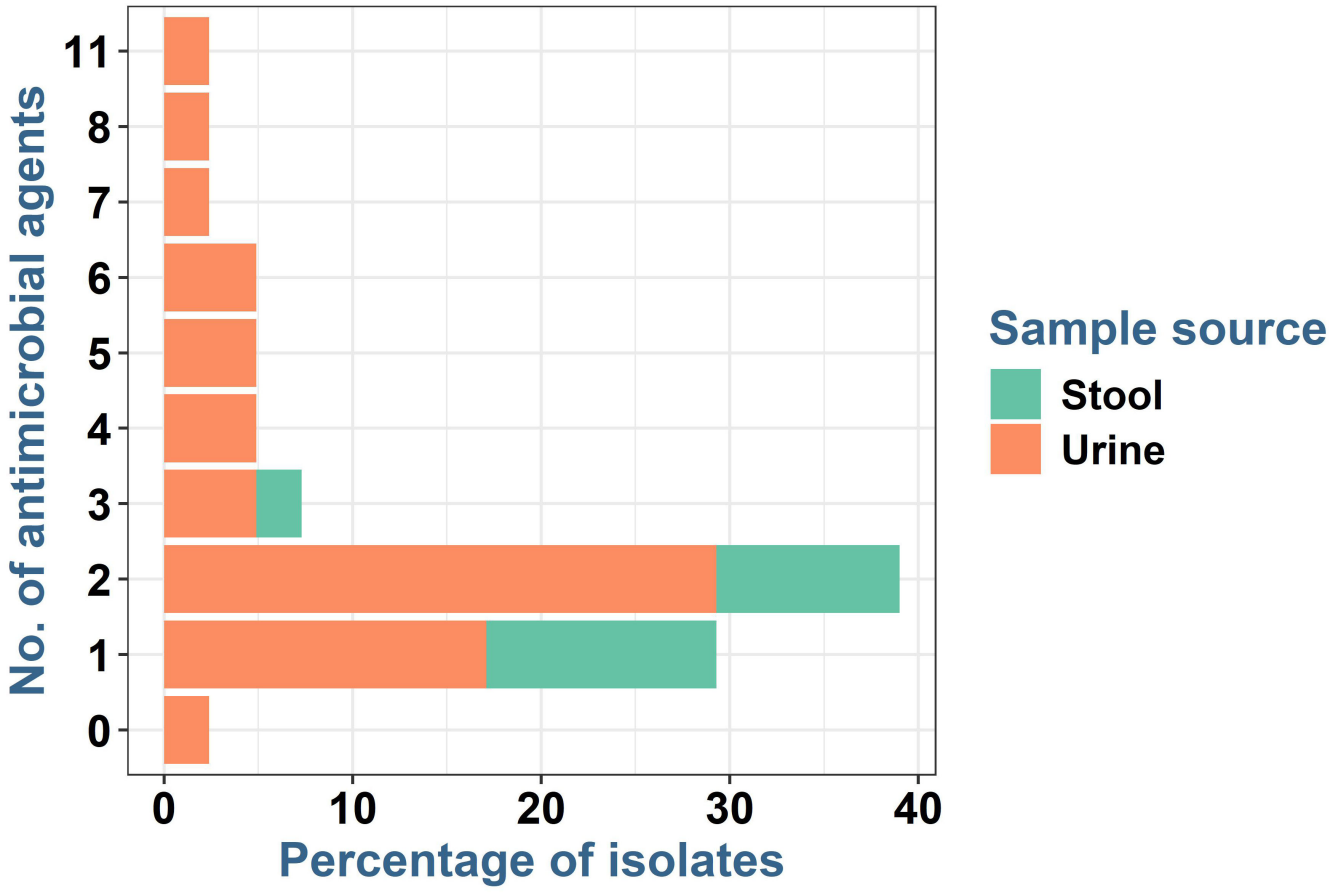
Frequency of resistance to various antimicrobial agents in *E. coli* isolated from human urine and stool samples.

*E. coli* isolates (*n* = 41) were resistant to at least one antibiotic (**Table 1**; **Figure 3**). The majority of the isolates (16, 39%) were resistant to two antibiotics, while resistance to four to six antibiotics was observed in two isolates each (4.9%, each). One isolate exhibited resistance to 11 antibiotics (2.4%). The MAR index of the isolates ranged from 0.08 to 0.85 with an average of 0.5.

**Table 1:** Frequency of resistance to various antimicrobial agents in *E. coli* isolates belonging to various sources.

MAR index	AMA	No. of *E. coli* isolates (%)		*P*-value	Total no. of *E. coli* isolates (%) (*n* = 41)
		Urine (*n* = 31)	Stool (*n* = 10)		
0	0	1 (3.2)	0	1	1 (2.4)
0.08	1	7 (22.6)	5 (50)	0.227	12 (29.3)
0.15	2	12 (38.7)	4 (40)	1	16 (39)
0.23	3	2 (6.5)	1 (10)	1	3 (7.3)
0.31	4	2 (6.5)	0	1	2 (4.9)
0.38	5	2 (6.5)	0	1	2 (4.9)
0.46	6	2 (6.5)	0	1	2 (4.9)
0.54	7	1 (3.2)	0	1	1 (2.4)
0.62	8	1 (3.2)	0	1	1 (2.4)
0.85	11	1 (3.2)	0	1	1 (2.4)

MAR, multiple antibiotic resistance; AMA, antimicrobial agents.

**Figure 4:**
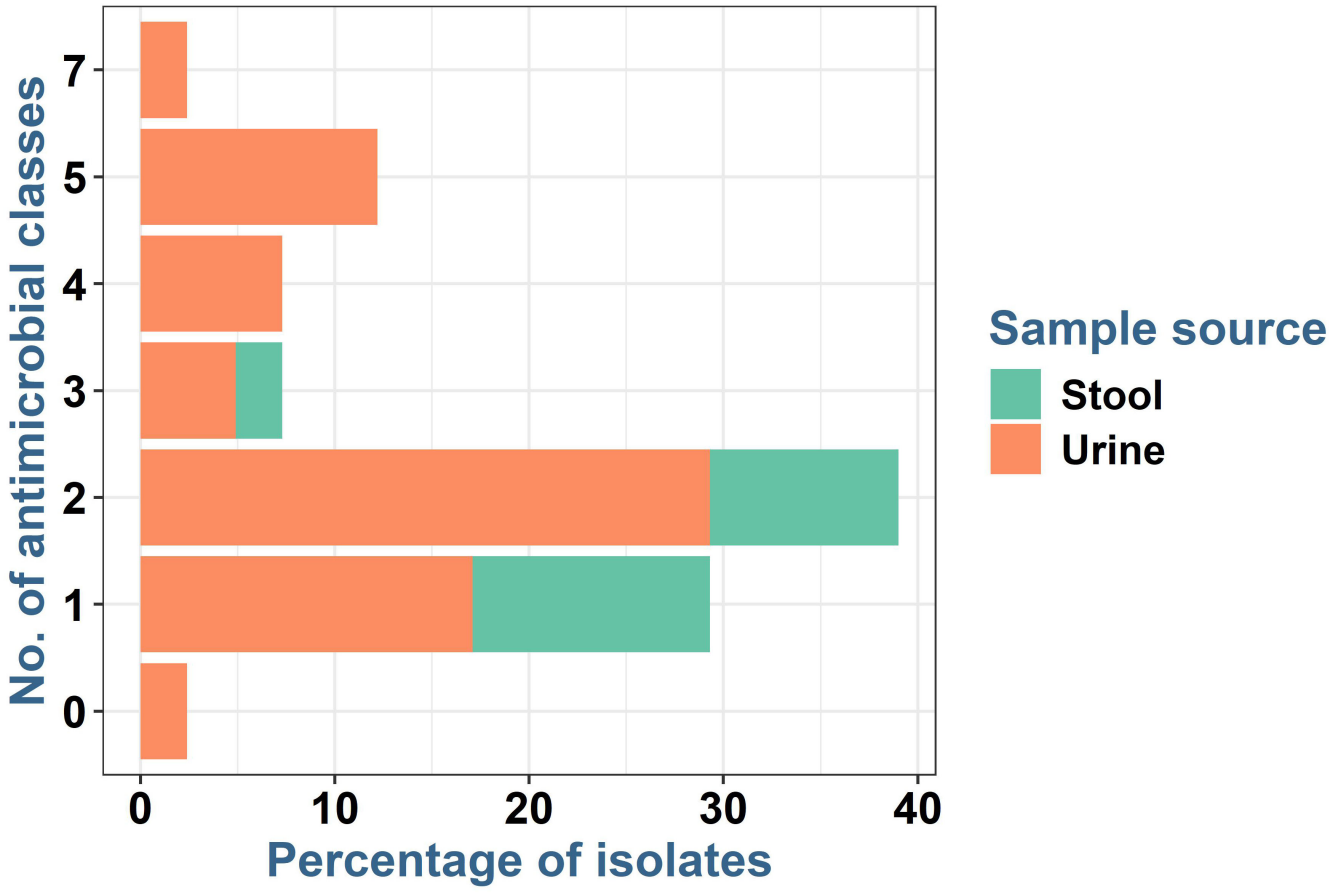
Frequency of resistance to various antimicrobial classes in *Escherichia coli* isolated from human urine and stool samples.

The results in **Figure 4** show that the majority of the *E. coli* isolates (9/10, 90%) from stool were resistant to antimicrobials in one (5, 50%) or two (4, 40%) classes, while only one isolate was resistant to antibiotics in three antimicrobial classes (10%). Multiple drug resistance was identified in six isolates from urine (19.3%) and one isolate from stool (10%). Extensive drug resistance was observed in six *E. coli* isolates from urine (14.6%) and one from stool samples (3.2%). There were no statistically significant differences (*p* > 0.05) in the frequencies of multiple drug resistance and extensive drug resistance among *E. coli* isolates from urine and stool samples.

Estimating the MAR indices for *E. coli* isolates revealed that 34.1% (12 out of 41) had an index > 0.2 (**Table 1**). Out of the 12 isolates, 11 (91.7%) were from urine origin, and one isolate from urine had a high MAR index of 0.86.

ESBL production of 41 *E. coli* isolates was examined phenotypically by PCDDT and DDST. The results revealed that PCDDT identified 20 (48.78%) isolates as ESBL-producing *E. coli*, whereas the DDST identified 13(31.7%) isolates. ESBL-producing *E. coli* were identified in 13 (41.9%) isolates from urine samples and in only 7 (17.1%) isolates from stool samples.

**Figure 5:**
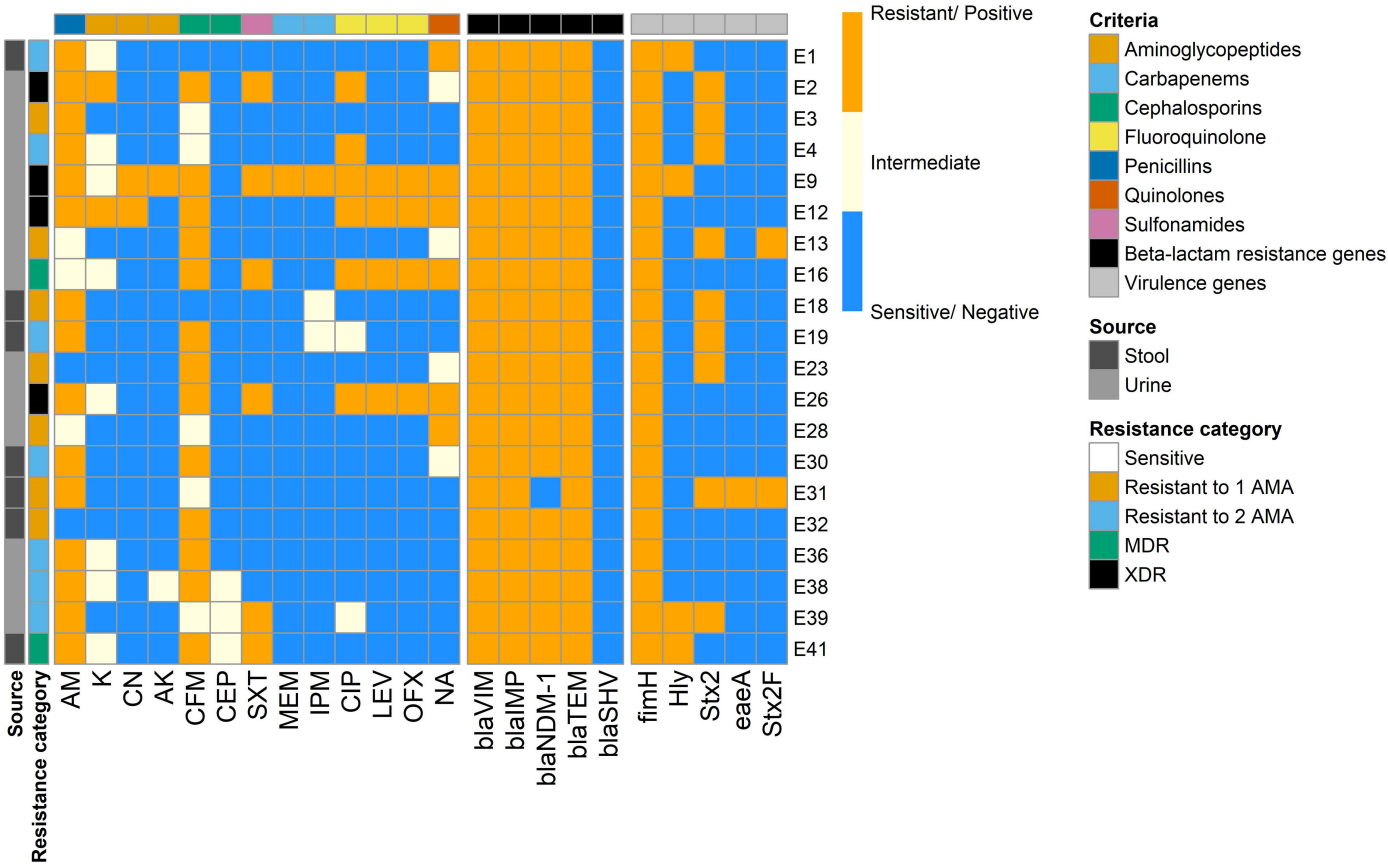
Heatmap showing the overall distribution of the investigated ESBL-producing *Escherichia coli* isolates based on the phenotypic antimicrobial resistance pattern, β-lactam resistance genes, and virulence genes. Different sample sources, resistance categories, isolate pathotypes, phylotypes, serotypes, and antimicrobial classes are color-coded on the right of the heatmap. ETEC, Enterotoxigenic *E. coli*; EPEC, Enteropathogenic *E. coli*; EIEC, Enteroinvasive *E. coli*; EHEC, Enterohemorrhagic *E. coli*; MDR, multidrug-resistant; XDR, extensive drug-resistant; AM, Ampicillin; S, Streptomycin; K, Kanamycin; CN, Gentamicin; AK, Amikacin; CFM, Cefixime; CEP, Cefoperazone; SXT, Trimethoprim/sulfamethoxazole; MEM, Meropenem; IPM, Imipenem; CIP, Ciprofloxacin; LEV, Levofloxacin; OFX, Ofloxacin; NA, Nalidixic acid; *bla*, β-lactamase; *fimH,* type 1 fimbriae D-mannose specific adhesion; *hly*, hemolysin; *Stx2*, Shiga toxin 2 subunit; *eaeA,* intimin adherence protein.

**Figure 6:**
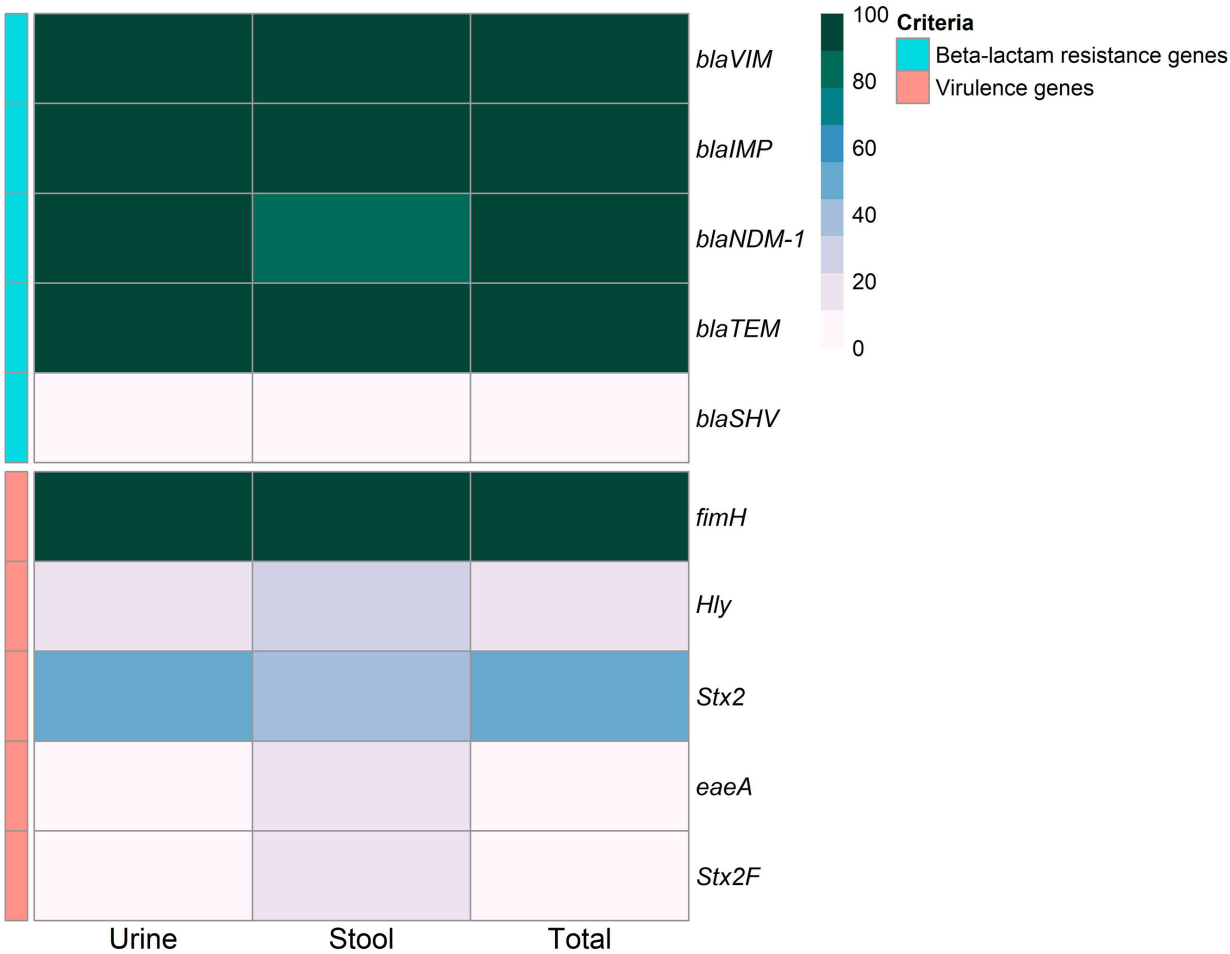
Frequency of extended-spectrum beta-lactamases production associated genes, and virulence genes in ESBL-producing *Escherichia coli* isolates. *bla*, β-lactamase; *fimH,* type 1 fimbriae D-mannose specific adhesion; *hly*, hemolysin O precursor; *Stx2*, Shiga toxin 2 subunit; *eaeA,* intimin adherence protein.

Phenotypic confirmed ESBL-producing *E. coli* isolates (*n* = 20) were subjected to molecular identification of ESBL-production-associated genes. PCR amplification produced amplicons corresponding to *bla*VIM, *bla*IMP, and *bla*TEM (**Figures 5**, **6**). Only one isolate was negative for NDM-1 gene (5%), while all the isolates were negative for *bla*SHV. There were no statistically significant differences in the detection rate of *bla*NDM-1 gene among ESBL-producing *E. coli* isolates recovered from human urine and stool samples (*p* = 0.35).

The phylogenetic grouping of 20 ESBL-producing *E. coli* showed that 19 (95%) isolates belonged to group B1, while only one isolate from urine samples (5%) belonged to group A. None of the isolates belonged to group B2 or D.

The genes encoding the virulence determinants, fimbrial adhesives (*fim*H), hemolysin O precursor (*hly*), Shiga toxin 2 subunit (*Stx2*), intimin adherence protein (*eae*A), and (*Stx*2F) were identified in the examined isolates by PCR (**Figures 5**, **6**). The results revealed that all the examined isolates harbored the *fim*H gene, while the *Stx2* gene was recorded in nine (45%) isolates. Four (20%) isolates were positive for the *hly* gene, whereas only one (5%) harbored the *eaeA* gene. The *Stx2F* gene was found in two isolates (10%) that were positive for the *Stx2* gene.

Out of 13 isolates from urine, 2 (6.5%) harbored the *hly* gene, 6 (19.4) were positive for the *Stx2* gene, while only 1 isolate (3.2%) harbored *Stx2F* gene (**Figures 5**, **6**). The isolates from stool samples (*n* = 7) were also positive for *hly* and *Stx2* genes with percentages of 28.5% (2 out of 7) and 42.9% (3 out of 7), respectively, while one isolate was positive for *Stx2F* and *eaeA* genes (14.3%, each). There were no statistically significant differences in the frequencies of *hly*, *stx*2, *eae*A, and *stx2*F genes among ESBL-producing *E. coli* isolated from urine and stool samples (*p* = 0.439, 1, 0.35, and 1, respectively).

**Figure 7:**
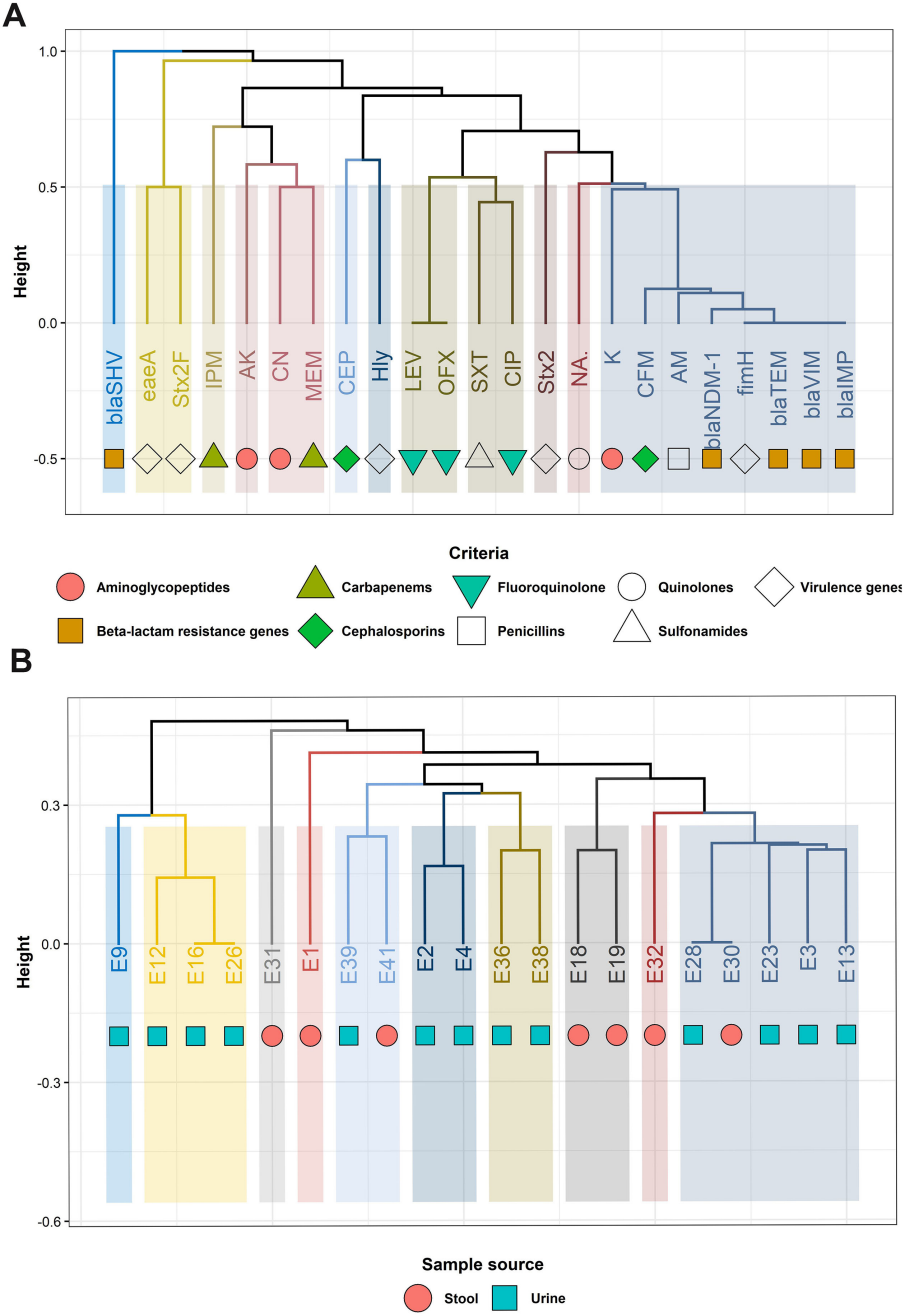
Hierarchical clustering dendrogram displaying the relatedness of the investigated variables **(A)**, ESBL-producing *Escherichia coli* isolates **(B)** as determined by the phenotypic antimicrobial resistance, β-lactam resistance genes, and virulence genes. AM, Ampicillin; S, Streptomycin; K, Kanamycin; CN, Gentamicin; AK, Amikacin; CFM, Cefixime; CEP, Cefoperazone; SXT, Trimethoprim/sulfamethoxazole; MEM, Meropenem; IPM, Imipenem; CIP, Ciprofloxacin; LEV, Levofloxacin; OFX, Ofloxacin; NA, Nalidixic acid; *bla*, β-lactamase; *fimH,* type 1 fimbriae D-mannose specific adhesion; *hly*, hemolysin O precursor; *Stx2*, Shiga toxin 2 subunit; *eaeA,* intimin adherence protein.

In the current study, **Figure 7** displays the relatedness of the investigated variables, ESBL-producing *E. coli* isolates as determined by the phenotypic AMR, β-lactam resistance genes, and virulence-associated genes. The investigated phenotypic AMR, β-lactam resistance genes, and virulence-associated genes displayed 12 branches and five clusters (**Figure 7A**). Among the 20 examined isolates, 16 isolates belonged to various lineages. Ten branches with six clusters were observed. Clustering analysis grouped β-lactam resistance genes and virulence-associated genes in the isolates from urine and stool samples (**Figure 7B**).

**Figure 8:**
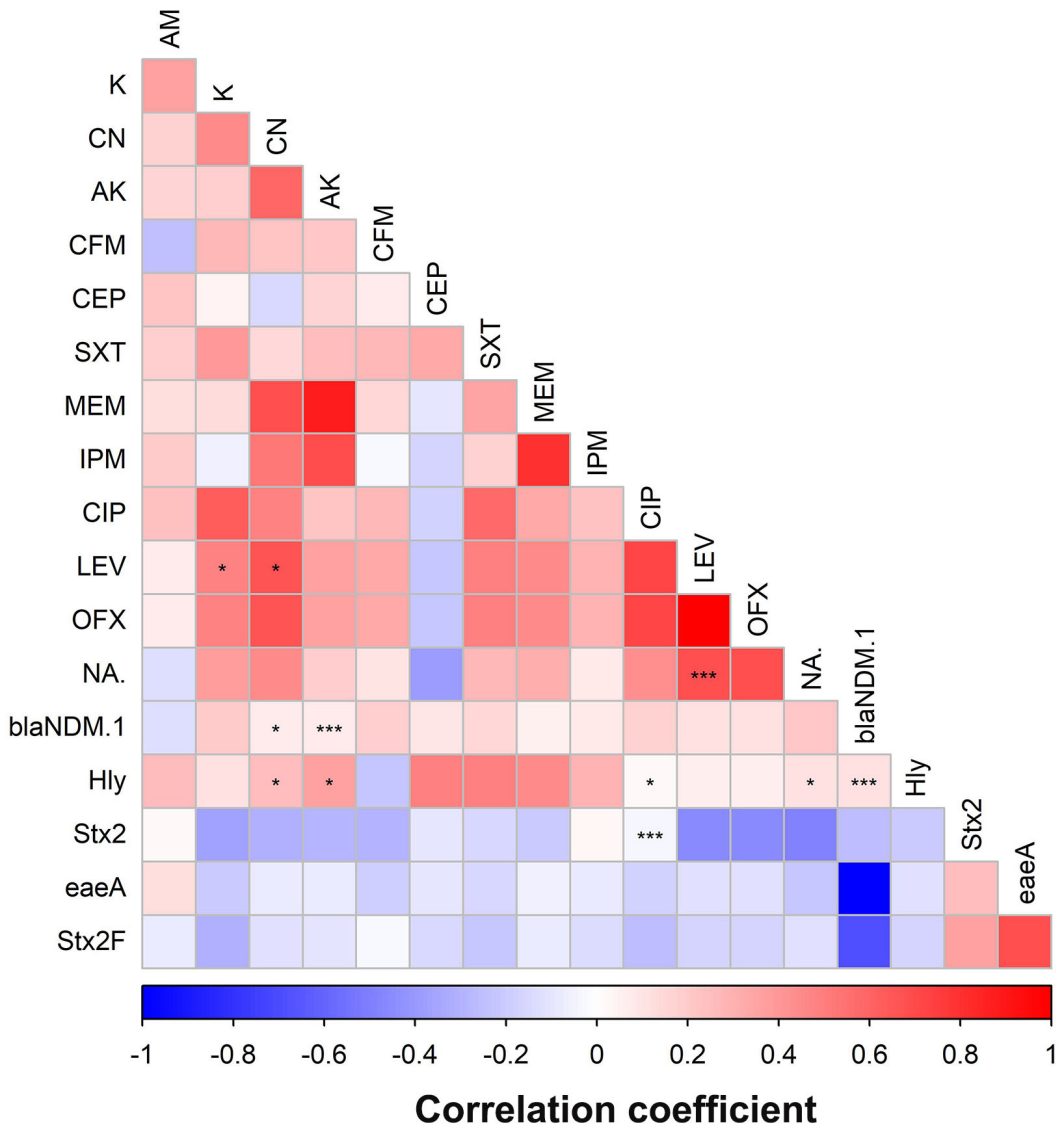
Pairwise correlation (*r*) between phenotypic antimicrobial resistance, β-lactam resistance genes, and virulence genes of ESBL-producing *Escherichia coli* isolates. The scale below the figure refers to the correlation coefficient (*r*). The more intense the color, the more the stronger the positive or negative correlation. Statistical significance was determined using Benjamini–Hochberg false discovery rate (FDR)–adjusted p-values to account for multiple comparisons. Asterisks indicate significant correlations (*adjusted *p* < 0.05, *** adjusted *p* < 0.001). Exact (*r*) values are provided in **Supplementary Figure 1**. Variables that are identical among all strains are excluded and thus not shown in this figure.

The results in **Figure 8** and **Supplementary Figure 1** show the correlation between phenotypic AMR and the presence of beta-lactam resistance genes and virulence-associated genes. A statistically significant positive correlation was observed between *Stx2*F and *eaeA* genes (*r* = 1, *p*-value < 0.0001) as well as a significant (*p* < 0.05) positive correlation between the *hly* gene and CEP, SXT, and MEM phenotypes (*r* = 0.49, 0.49, and 0.46, respectively). However, the *bla*NDM-1 gene correlated significantly and negatively with the *Stx*2F gene (*r* = −1, *p*-value < 0.0001) and *eaeA* (*r* = −688, *p*-value = 0.001). A significant (*p* < 0.05) negative correlation between the *Stx*2 gene and LEV, OFX, and NA phenotypes (*r* = −0.45, −0.45, and −0.48, respectively) was also observed.

**Figure 9:**
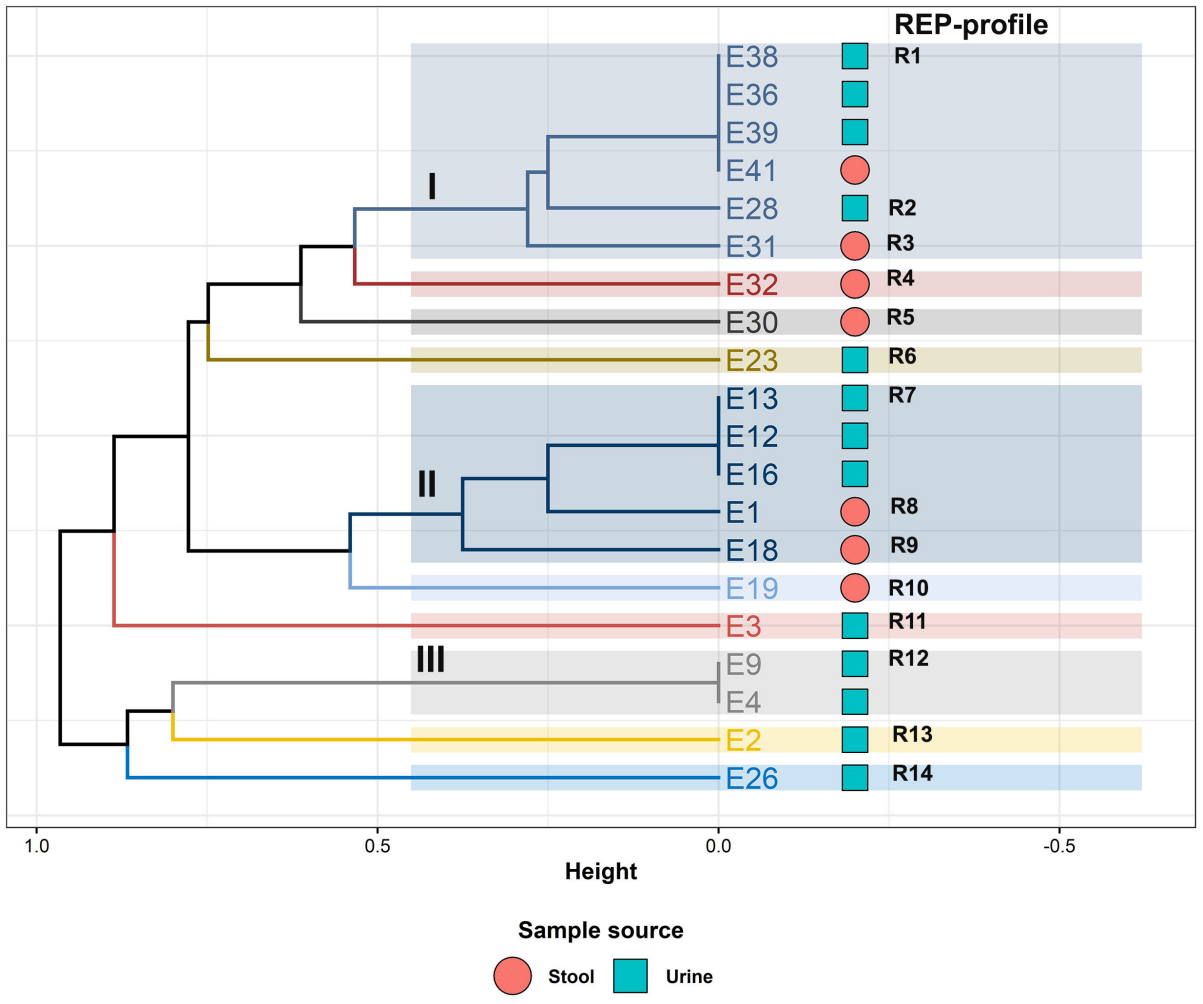
Hierarchical clustering dendrogram showing the relatedness of ESBL-producing *Escherichia coli* isolates from urine and stool samples as determined by REP-PCR fingerprinting.

In the current study, REP-PCR was utilized to determine the genetic relatedness of different ESBL-producing *E. coli* isolates from urine and stool samples. The results revealed that REP-PCR classified ESBL-producing *E. coli* strains into 14 distinct profiles (R1–R14), and the amplicon sizes ranged from 200 to 3,400 bp (**Table 2**; **Figure 9**).

**Table 2:** REP-PCR fingerprinting profile and associated clusters for ESBL-producing *Escherichia coli* recovered from urine and stool samples.

No. of isolates	Profile	Source	Isolate code	Cluster
4	R1	Urine	E38	I
		Urine	E36	
		Urine	E39	
		Stool	E41	
1	R2	Urine	E28	
1	R3	Stool	E31	
3	R4	Urine	E13	II
		Urine	E12	
		Urine	E16	
1	R5	Stool	E1	
1	R6	Stool	E18	
2	R7	Urine	E9	III
		Urine	E4	
1	R8	Stool	E32	Single isolates
1	R9	Stool	E30	
1	R10	Urine	E23	
1	R11	Stool	E19	
1	R12	Urine	E3	
1	R13	Urine	E2	
1	R14	Urine	E26	

The hierarchical clustering (HA) dendrogram analysis of the examined isolates (*n* = 20) showed 10 REP branches with three clusters (I, II, and III) and seven separate isolates (**Table 2**; **Figure 9**). REP-PCR showed a Simpson Discriminatory index (*D*) of 0.947. Three REP profiles (R1, R7, and R12) showed clusters of two to four identical isolates per REP profile.

The SeNPs were biosynthesized by *A. fumigatus.* For identification of species, molecular characterization was conducted using the 18S–28S rRNA gene sequence. DNA was obtained from the *Aspergillus* species. The 18S–28S rRNA gene was subjected to the PCR test, where PCR content revealed a DNA band with a size of approximately 569 bp and the 18S–28S rRNA sequence bordering the ITS1, 2, and 5.8 S regions for *Aspergillus* sp. The fungal isolates have been identified as *Aspergillus* sp. with accession number MT321105 by a combination of morphological and molecular examinations. From the alignment profile, *Aspergillus* sp. (sample 1), for numerous strains of the same species containing the type material *A. fumigatus* (ATCC1022, gb: NR121481), this strain demonstrated 100% identity. The isolate exhibited in this investigation matched with sequences from the GenBank that were quite similar to it. Therefore, it was developed as *A. fumigatus-*FM, AUMC14445 gb (569 letters); it was patterned as *A. fumigatus* with accession no. MT321105 (**Supplementary Figure 2**).

**Figure 10:**
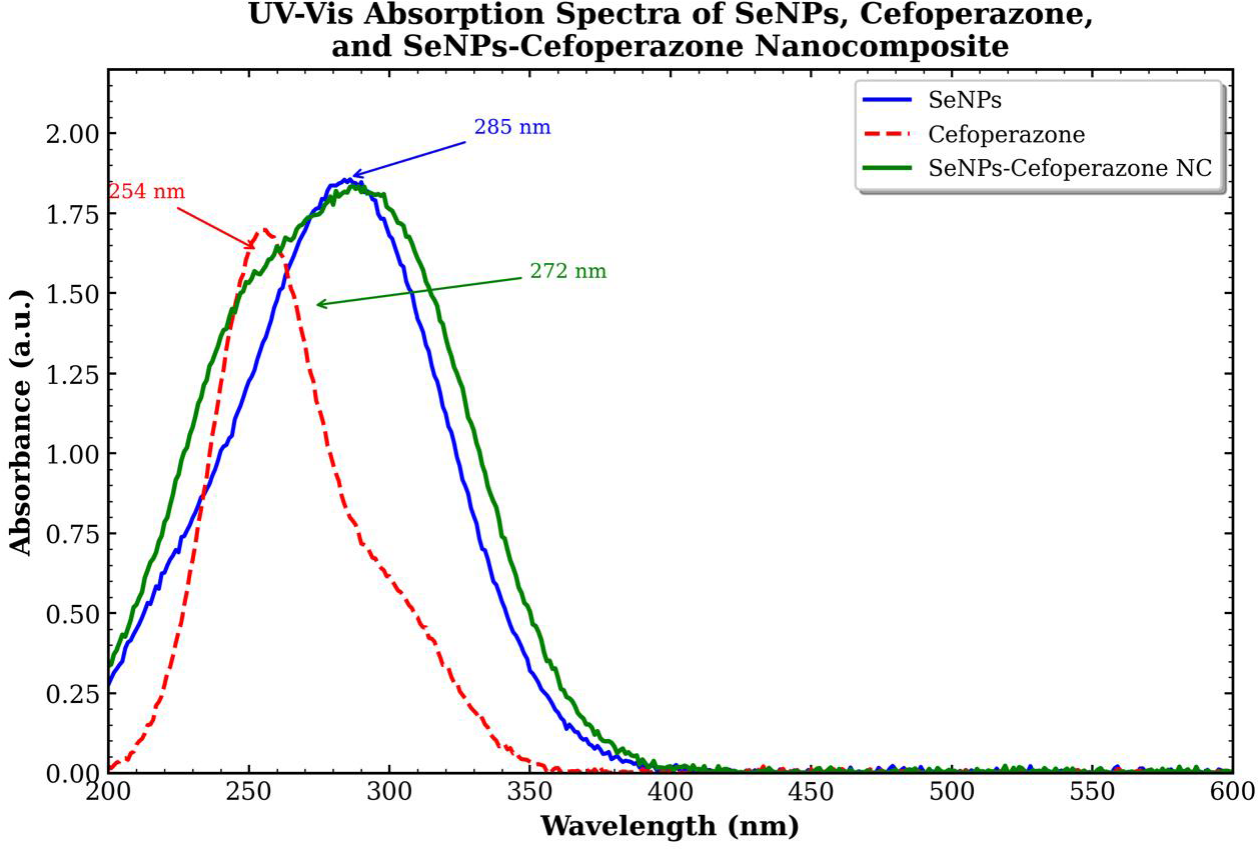
UV–Vis spectroscopy for SeNPs, cefoperazone, and SeNCs (SeNPs–cefoperazone nanocomposite).

SeNPs exhibit a characteristic SPR absorption band in the 265- to 350-nm regions, arising from collective oscillation of conduction electrons. CEP, a third-generation cephalosporin antibiotic, shows strong absorption at approximately 254 nm due to its aromatic hydroxyphenyl ring, conjugated amide system, and tetrazole heterocycle. The SeNCs spectrum reveals merged contributions from both components with characteristic shifts indicating successful conjugation. The absorption spectra were analyzed for peak position, peak intensity, full width at half maximum (FWHM), and band gap energy. The optical band gap was estimated using the Tauc plot method. Peak shifts between bare SeNPs and the SeNCs were calculated to confirm surface modification and drug conjugation. The red shift of approximately 13 nm from bare SeNPs to the SeNCs SPR peak indicates successful surface interaction between CEP and the SeNPs surface (**Figure 10**).

**Figure 11:**
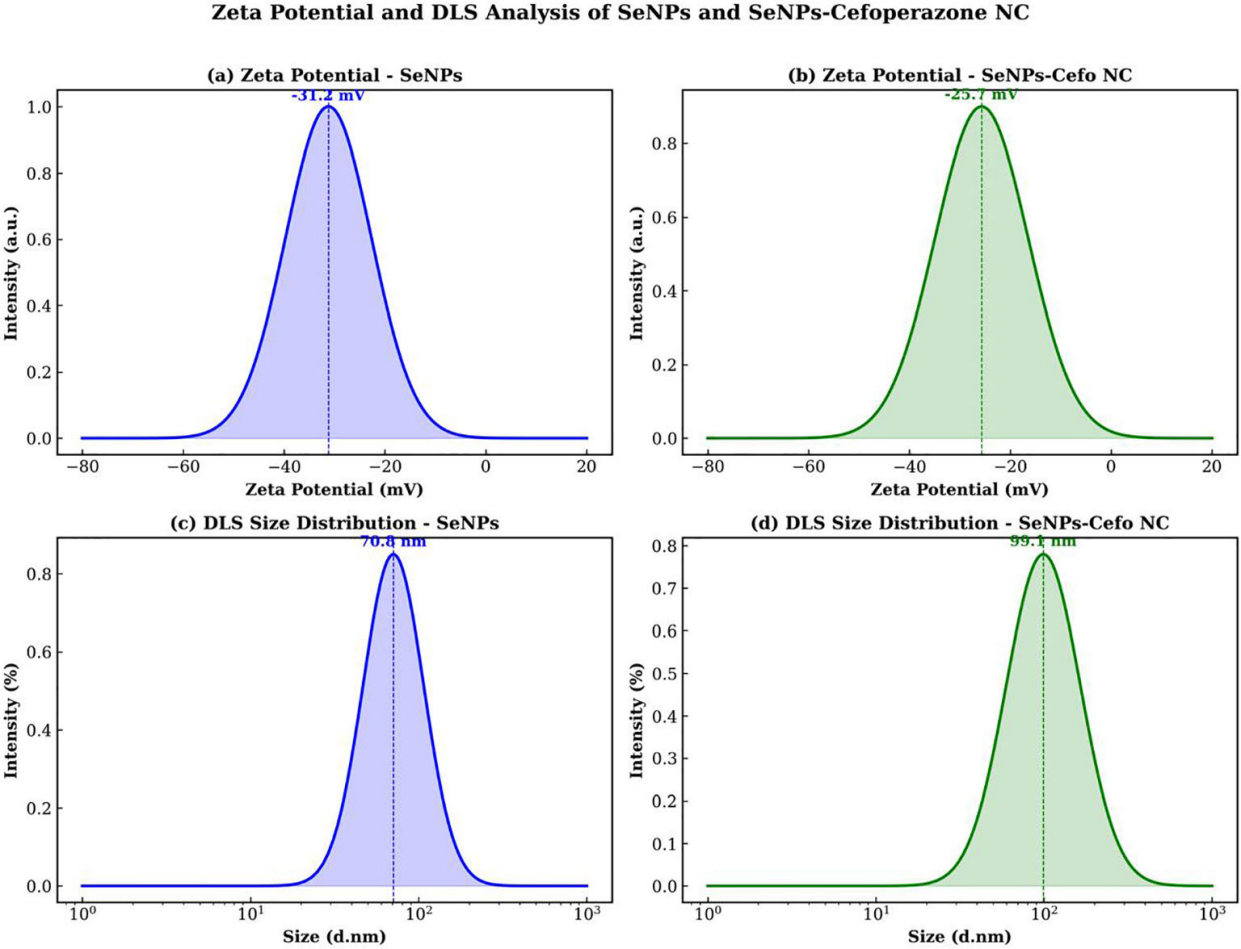
Zeta potential and dynamic light scattering (DLS) characterization of nanoparticles. **(A)** Zeta potential of SeNPs. **(B)** Zeta potential of SeNCs. **(C)** DLS diameter of SeNPs. **(D)** DLS diameter of SeNCs.

The zeta potential of bare SeNPs was measured as −31.2 mV, indicating good colloidal stability attributed to the negatively charged biological capping agents (proteins and polysaccharides) from the fungal extract (**Figure 11A**). Upon conjugation with CEP, the zeta potential shifted to −25.7 mV (a reduction of 5.5 mV in magnitude) (**Figure 11B**). This modest shift is consistent with partial surface neutralization due to drug adsorption while maintaining adequate stability. The DLS hydrodynamic diameter increased from 70.82 nm (SeNPs) to 99.1 nm (nanocomposite), confirming the formation of a drug corona around the SeNP core (**Figures 11C, D**). The PDI increase from 0.218 to 0.312 reflects the broadened size distribution expected upon conjugation (**Table 3**).

**Table 3:** Zeta potential and dynamic light scattering for SeNPs and SeNCs.

Parameter	SeNPs	SeNCs
Zeta potential (mV)	−31.2 ± 1.8	−25.7 ± 2.1
*Z*-average (nm)	70.8 ± 3.2	99.1 ± 5.8
PDI	0.218 ± 0.015	0.312 ± 0.024
Conductivity (mS/cm)	0.245	0.312
Stability	Good	Moderate-good

**Figure 12:**
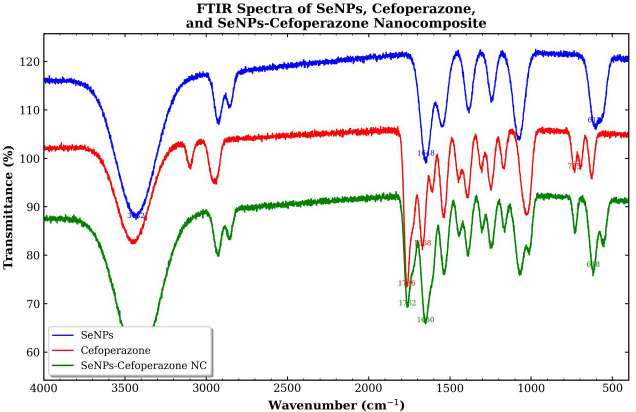
Fourier transform infrared (FTIR) spectroscopy for SeNPs, cefoperazone, and SeNCs (SeNPs–cefoperazone nanocomposite).

FTIR spectra confirmed the successful formation of SeNPs and their interaction with CEP in the nanocomposite (SeNCs). The broad O–H/N–H stretching band appeared at 3,432 cm^−1^ for SeNPs and 3,448 cm^−1^ for CEP, while it shifted to 3,418 cm^−1^ in the SeNCs, indicating hydrogen bonding interactions between the drug and nanoparticle surface. The characteristic β-lactam carbonyl (C=O) band of CEP was observed at 1,766 cm^−1^ and remained nearly unchanged at 1,762 cm^−1^ in the SeNCs, confirming that the pharmacophoric β-lactam ring remained intact after loading. The amide I band shifted from 1,668 cm^−1^ (CEP) to 1,650 cm^−1^ in the SeNCs, compared to 1,648 cm^−1^ in SeNPs, suggesting surface coordination between CEP and SeNPs. Additionally, the Se–O stretching vibration appeared at 612 cm^−1^ in SeNPs and shifted to 618 cm^−1^ in the SeNCs, confirming selenium incorporation. The C–S stretching band of CEP at 735 cm^−1^ slightly shifted to 730 cm^−1^ in the SeNCs, indicating the involvement of the sulfur-containing ring in the interaction (**Figure 12**).

**Figure 13:**
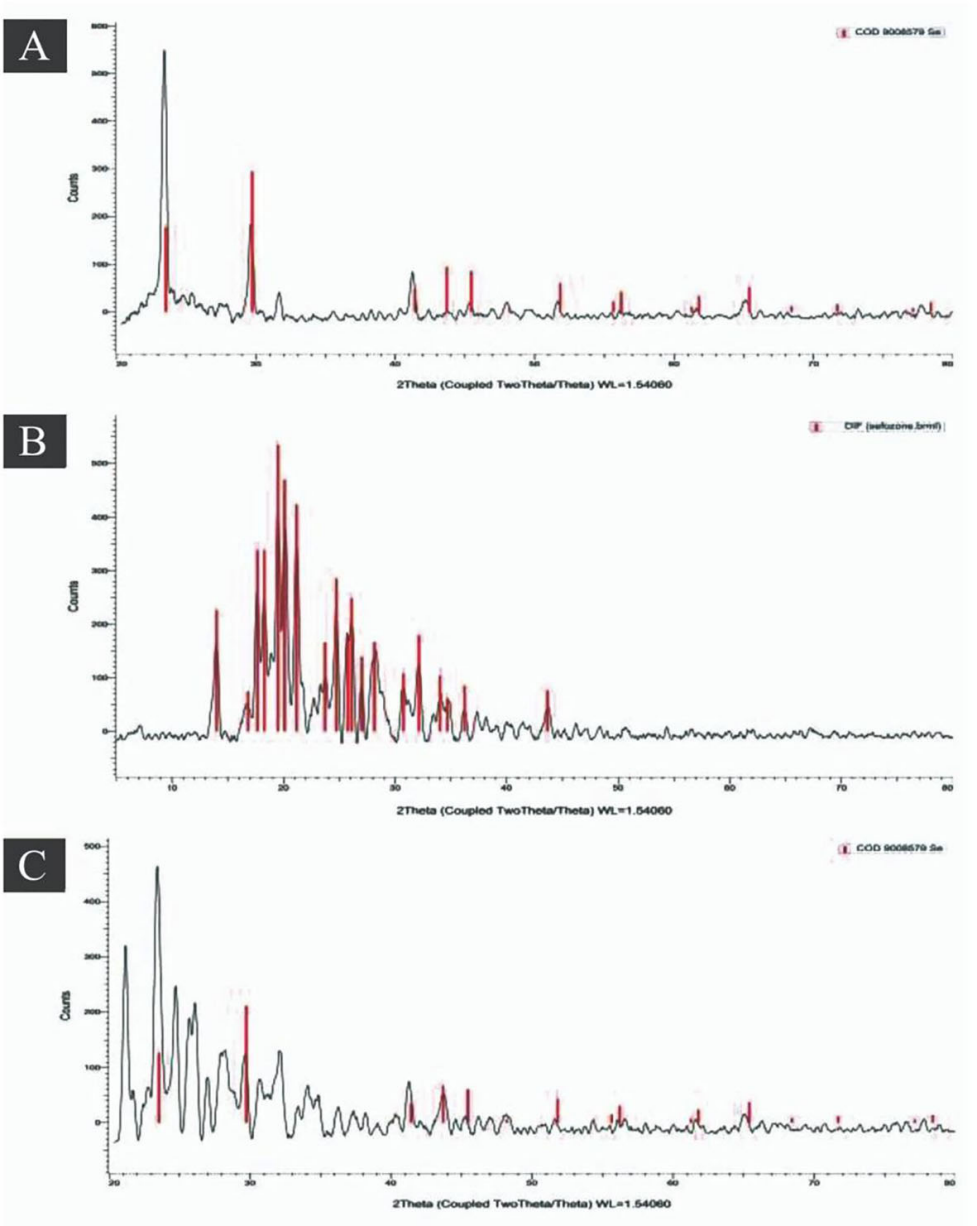
Powder x-ray diffraction (XRD) pattern for **(A)** SeNPs, **(B)** cefoperazone (CEP), and **(C)** SeNCs.

The x-ray diffraction of SeNPs, antibiotic, and SeNCs are given in **Figure 13**. A feature area at a 2θ angle of 18.664°, 23.943°, 31.306°, 37.306°, 42.874°, 47.743°, 48.016°, 67.399°, and 78.164° was noticed for SeNPs (**Figure 13A**). In addition, a feature area at a 2θ angle of 18.664°, 23.943°, 31.306°, 37.306°, 42.874°, 47.743°, 48.016°, 67.399°, and 78.164° were noticed for CEP (**Figure 13B**). Furthermore, SeNCs appeared in major characteristic peaks at 2θ angles corresponding to 18.321°, 23.182°, 25.618°, 29.917°, 30.777°, 33.340°, 42.841°, 53.442°, 63.896°, 65.119°, and 75.803° (**Figure 13C**).

**Figure 14:**
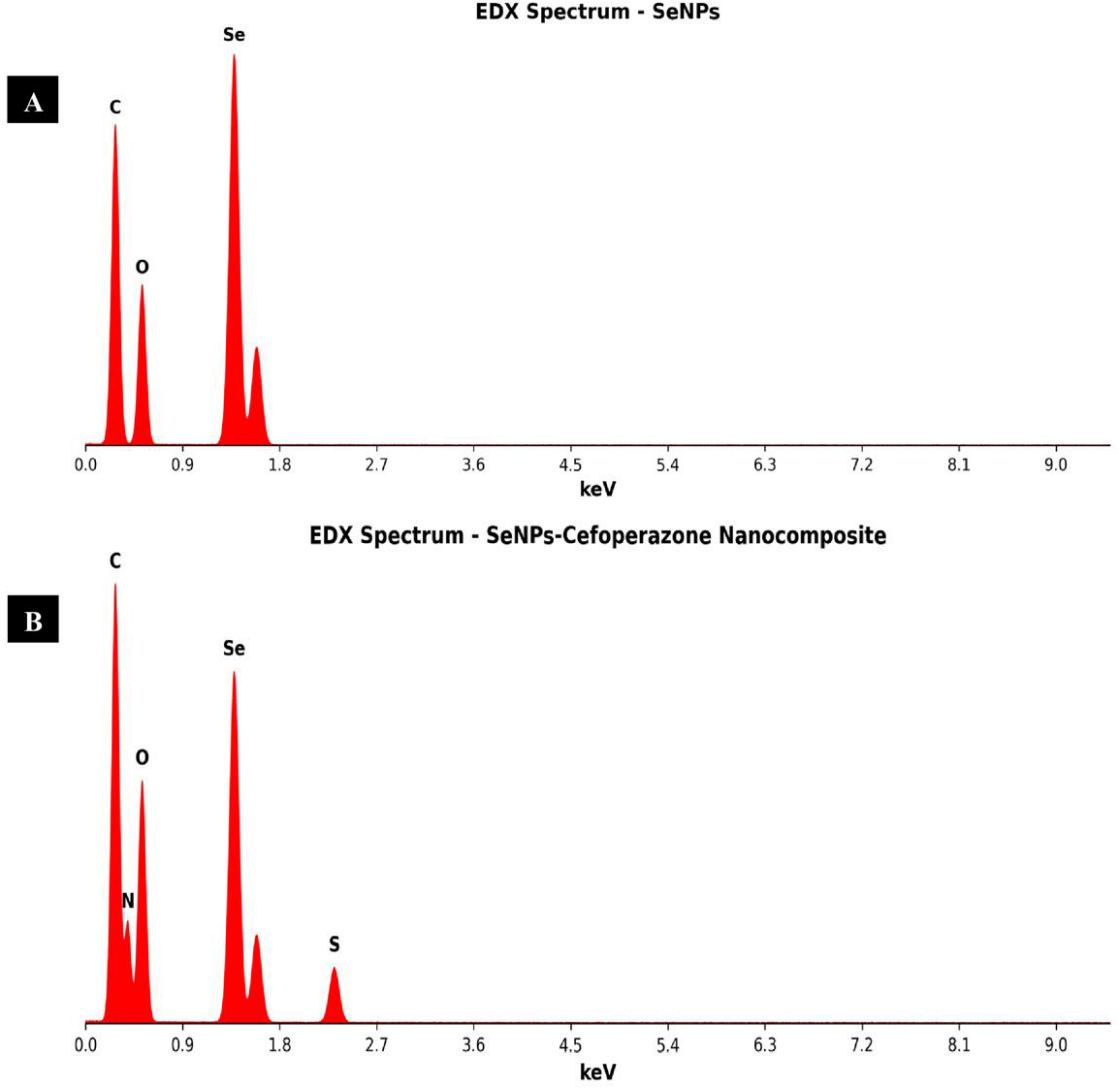
EDX pattern for **(A)** SeNPs and **(B)** SeNCs.

The EDX spectrum of bare SeNPs shows dominant selenium peaks (Se La at 1.379 keV) comprising 72.45 wt%, with carbon (16.83 wt%) and oxygen (10.72 wt%) from the biological capping agents derived from the fungal extract (**Figure 14A**). Upon conjugation with CEP, the Se content decreases to 45.32 wt% due to the organic drug shell. The appearance of nitrogen (8.92 wt%) and sulfur (3.75 wt%) peaks in the nanocomposite spectrum provides definitive evidence of CEP incorporation (**Figure 14B**). Sulfur is particularly diagnostic as it originates exclusively from CEP (two sulfur atoms per molecule in the thioether and dihydrothiazine moieties). The N-to-S atomic ratio of approximately 7.8:1 reflects contributions from both CEP (theoretical 9N:2S = 4.5:1) and additional nitrogen from biological capping proteins.

The data exhibited spherical forms for SeNPs by TEM (**Supplementary Figure 2A**). Also, SeNCs exhibited sub-rectangular forms (**Supplementary Figure 2B**). TEM imaging reveals spherical nanoparticles with mean diameters of 70.8 nm for bare SeNPs and 99.1 nm for the nanocomposite. The size increase of approximately 28.3 nm upon drug conjugation is consistent with the formation of a CEP corona (estimated 14 nm thickness) around the selenium core. The nanocomposite particles exhibit a core–shell morphology with a higher electron-density selenium core surrounded by a lower-contrast organic shell visible in HR-TEM images.

The antibacterial effect of SeNPs and SeNCs against isolates of *E. coli* recovered from stool and urine specimens was assessed using the well diffusion method (**Table 4**). All isolates (100%) were sensitive to CEP that was used as a positive control. SeNPs and SeNCs produced significantly larger inhibition zones compared to CEP with all *E. coli* isolates. Furthermore, the MIC value required to inhibit visible growth of *E. coli* was 10 µg/mL for SeNCs and 20 µg/mL for SeNPs (**Table 5**).

**Table 4:** Antibacterial activity and inhibition zone diameter (mm) of 100 µg/mL concentration of cefoperazone (CEP), SeNPs, and SeNCs against *E. coli* isolates.

Strains	Cefoperazone (CEP)	SeNPs	SeNCs
E1 s	27.80 ± 0.30 a^,c *^	33.63 ± 0.40 a^,b,c *^	39.76 ± 0.40 a^,b,c *^
E4 u	24.93 ± 0.93 a^,c *^	33.73 ± 0.31 a^,b *^	43.63 ± 0.35 a^,b,c *^
E6 s	26.56 ± 0.38 a ^*^	32.30 ± 0.26 a^,b *^	42.56 ± 0.21 a^,b,c *^
E7 s	28.56 ± 1.27 a ^*^	35.76 ± 0.59 a^,b *^	42.86 ± 0.32 a^,b,c *^
E9 u	22.03 ± 0.95 a^,c *^	28.66 ± 0.35 a^,b,c *^	43.16 ± 0.67 a^,b,c *^
E14 u	26.63 ± 0.40 a ^*^	31.73 ± 0.42 a^,b *^	45.30 ± 0.66 a^,b,c *^
E15 u	22.16 ± 0.80 a^,c *^	33.93 ± 0.21 a^,b *^	43.76 ± 0.25 a^,b,c *^
E18 s	29.56 ± 1.27 a ^*^	32.66 ± 0.45 a^,b,c *^	42.83 ± 0.32 a^,b,c *^
E19 s	23.33 ± 0.87 a^,c *^	33.76 ± 0.31 a^,b *^	43.96 ± 0.49 a^,b,c *^
E21 u	27.20 ± 0.80 a ^*^	33.10 ± 0.30 a^,b *^	43.46 ± 0.61 a^,b,c *^
E26 u	22.23 ± 0.78 a^,c *^	35.20 ± 0.79 a^,b *^	44.60 ± 0.46 a^,b,c *^
E29 s	30.20 ± 1.22 a ^*^	30.30 ± 0.75 a^,c *^	42.70 ± 0.26 a^,b,c *^
E30 s	30.00 ± 0.92 a ^*^	32.50 ± 1.32 a^,b *^	41.83 ± 0.15 a^,b,c *^
E31 s	29.26 ± 1.06 a ^*^	35.23 ± 0.71 a^,b *^	42.80 ± 0.10 a^,b,c *^
E32s	30.83 ± 0.25 a^,c *^	32.10 ± 0.46 a^,b *^	39.90 ± 0.26 a^,b *^
E34 u	25.30 ± 0.50 a ^*^	34.10 ± 0.46 a^,b *^	41.26 ± 0.42 a^,b *^
E35 u	28.36 ± 0.55 ^a*^	33.40 ± 0.60 a^,b *^	40.66 ± 0.35 a^,b *^
E38 u	19.76 ± 1.10 a^,c *^	33.40 ± 0.72 a^,b *^	42.33 ± 0.65 a^,b,c *^
E39 u	29.23 ± 0.86 ^a*^	33.30 ± 0.80 a^,b *^	41.90 ± 0.17 a^,b,c *^
E41 s	17.30 ± 0.66 a^,c *^	31.50 ± 1.50 a^,b,c *^	43.56 ± 0.45 a^,b,c *^

*Statistically significant at *p* < 0.05 according to one-way ANOVA. a–c: Different letters within the same column indicate significant differences for pairwise comparisons between concentrations within each isolate at *p* < 0.05 according to *post-hoc* Tukey test.

**Table 5:** Minimum inhibition concentration (MIC) of cefoperazone (CEP), SeNPs, and SeNCs against *E. coli* isolates.

Tested sample	Concentration, µg/mL	E4 urine	E9 urine	E15 urine	E34 urine	E19 stool	E18 stool	E26 urine	E39 urine	E41 stool
CEP	5	+	+	+	+	+	+	+	+	+
	10	+	+	+	+	+	+	+	+	+
	20	_	_	_	_	_	_	_	_	_
	50	_	_	_	_	_	_	_	_	_
	100	_	_	_	_	_	_	_	_	_
SeNPs	5	+	+	+	+	+	+	+	+	+
	10	+	+	+	+	+	+	+	+	+
	20	_	_	_	_	_	_	_	_	_
	50	_	_	_	_	_	_	_	_	_
	100	_	_	_	_	_	_	_	_	_
SeNCs	5	+	+	+	+	+	+	+	+	+
	10	_	_	_	_	_	_	_	_	_
	20	_	_	_	_	_	_	_	_	_
	50	_	_	_	_	_	_	_	_	_
	100	_	_	_	_	_	_	_	_	_

−: No growth. +: positive growth.

**Figure 15:**
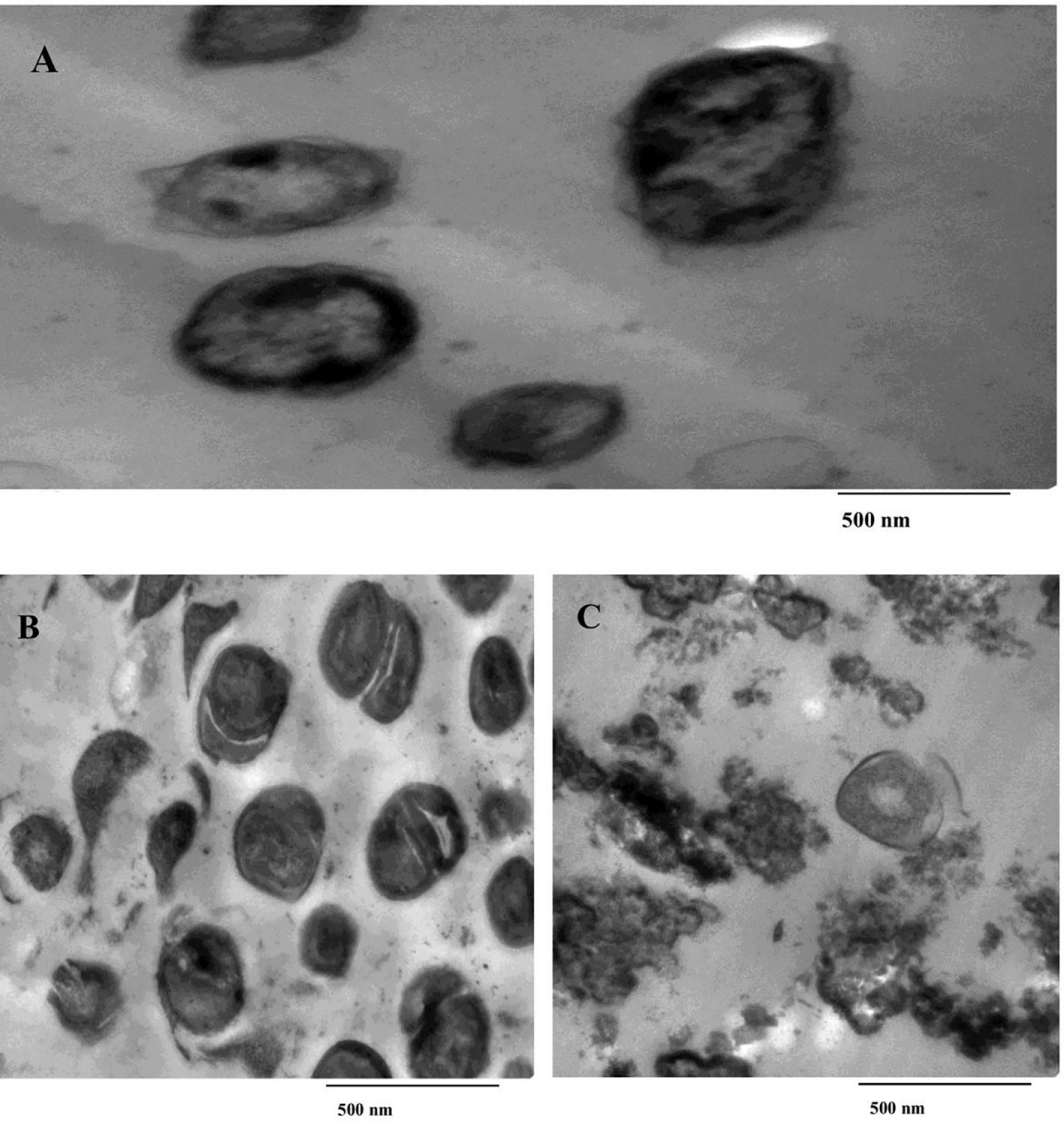
Transmission electron microscopy (TEM) images showing the antibacterial activity of selenium nanoparticles (SeNPs) and selenium nanocomposites (SeNCs) against *E. coli*. **(A)** Untreated *E. coli* cells (control). **(B)***E. coli* cells treated with SeNPs. **(C)***E. coli* cells treated with SeNCs.

The treatment of *E. coli* suspensions with 20 and 10 µg/mL, respectively, of purified SeNPs and SeNCs has resulted in a marked elevation in destroyed cells following their maintenance at 37°C, as revealed by TEM images in **Figure 15**. Images indicated the existence of alterations in *E. coli* treated with SeNPs or SeNCs. Cell deformation; adhesion to lysed cell material resulting in malformations, cell clumping, blisters, and cellular depressions; and decreased cell counts were seen in TEM images of *E. coli*. In addition, the formation of highly damaged cells was indicated with strange shapes and an empty central zone formerly occupied by the nucleoid using SeNCs (**Figure 15**).

## Discussion

This study investigated the molecular characteristics and antimicrobial profiles of ESBL-producing *E. coli* from patients with UTI and assessed the efficacy of selenium-based nanocomposites as alternative therapeutic agents.

The AMR profile observed in the current study reflects the persistent selective pressure resulting from the widespread use of β-lactam antibiotics in clinical settings. High resistance to AM and cephalosporins aligns with reports from various regions where β-lactam resistance among UTI *E. coli* isolates has become increasingly prevalent (Sabir et al., 2014; Sharma et al., 2020). Similar resistance trends, particularly against AM, CIP, and SXT, have been reported in India, where MDR patterns are frequently observed (Bernaitis et al., 2024).

The observed high activity of aminoglycosides in the present study is consistent with the findings of Odonkor and Addo (2018) who reported high sensitivity to AK and CN.

Elevated MAR indices (more than 0.2) among MDR isolates suggest that *E. coli* isolates are from a high-risk source of contamination, and they have been exposed to multiple antibiotics overtime (Khan et al., 2015). This continuous exposure results in selective pressure, leading to the survival and proliferation of MDR bacteria (Tello et al., 2012).

The higher frequency of MDR among urine isolates compared with stool isolates observed in this study is consistent with findings reported by Navidinia et al. (2014) and Norouzian et al. (2019) who reported higher AMR among urinary isolates. High susceptibility was observed for *E. coli* isolates from urine and stool to IPM, thus demonstrating the high activity of IPM against ESBL-producing *E. coli* (Navidinia et al., 2014; Norouzian et al., 2019). This is because IPM is among the most stable carbapenems against ESBLs and are commonly preferred for treating serious infections (Paterson, 2006).

The variations in AMR patterns of *E. coli* isolates across studies may reflect differences in geographic distribution, antibiotic administration practices, and local selective pressures (Ewers et al., 2012).

The detection of ESBL-producing *E. coli* in the current study highlights the continued dissemination of plasmid-mediated ESBL genes among clinical isolates. This, in turn, results in the emergence of MDR pathogens due to the rapid dissemination of ESBL genes across different species (Azab et al., 2021; Husna et al., 2023).

The frequencies of ESBL-producing *E. coli* vary across geographic regions, for instance, 4.1% in Japan (Hayami et al., 2019), 10% in Korea (Park et al., 2017), 2.2%–24% in Europe (Chervet et al., 2018), and 72.4% in Pakistan (Razaq et al., 2024). This variation could be attributed to population characteristics, antibiotic pressure, and treatment policies.

Molecular screening of ESBL-associated genes in the present study revealed a high frequency of *bla*VIM, *bla*IMP, and *bla*TEM. The predominance of these genes suggests the dissemination of plasmid-mediated resistance elements among clinical isolates. Comparable studies have reported varying distributions of ESBL-associated genes across geographic regions, with *bla*TEM and *bla*SHV frequently detected among UTI-associated isolates (Perera et al., 2022; Razaq et al., 2024). A high rate of ESBL-producing *E. coli* from stool samples (34.8%) was reported by Tellevik et al. (2016). The relatively high frequencies of ESBLs in *E. coli* isolates from stool samples of patients with UTI indicate that the presence of ESBL-producing *E. coli* in stool can be considered as a potential risk for UTI (Rodríguez-Baño et al., 2006).

The detection of *bla*VIM and *bla*IMP genes despite carbapenem susceptibility could be attributed to the low level of expression, gene mutations, or non-functional gene variants. Similar observations have been reported, and the authors suggested sequencing and expression analysis (Son et al., 2021).

It is important to note that this study employed PCR-based screening and did not include sequencing confirmation of the amplified products. Therefore, the presence of the target-sized PCR products should be interpreted as indicative of gene detection rather than definitive confirmation of gene expression and functional activity. Additionally, the absence of *bla*CTX-M screening, which is currently the most prevalent ESBL gene family, represents a limitation of the present study. Consequently, the molecular characterization presented here reflects the targeted detection of the selected β-lactamase genes rather than a comprehensive ESBL genotyping. Future studies incorporating *bla*CTX-M and other ESBL variants are warranted to provide a more complete epidemiological picture.

*E. coli* can be categorized into four principal phylogenetic groups (A1, B1, B2, and D) based on PCR amplification of specific genetic markers (Gordon et al., 2008). Traditionally, groups A1 and B1 include mainly commensal strains, while groups B2 and D are associated with extraintestinal pathogenic *E. coli*, including UPEC (Gordon et al., 2008; Martínez-Vázquez et al., 2022).

In the present study, out of 20 ESBL-producing *E. coli*, 19 (95%) isolates were assigned to phylogroup B1, while only one isolate (5%) belonged to group A; no isolates were classified as B2 or D. This distribution is atypical compared to the classical association of UPEC with phylogroup B2. However, increasing evidence suggests that AMR determinants, particularly plasmid-mediated ESBL genes, can disseminate across diverse phylogenetic groups strains (Ibekwe et al., 2021; Ullah et al., 2023). The acquisition of mobile genetic elements carrying resistance genes may enable traditionally commensal isolates, such as B1, to participate in extraintestinal infections.

Previous studies have similarly reported the predominance of commensal phylogroups among ESBL-producing isolates (Ohta and Ikeda, 2021; Martínez-Vázquez et al., 2022; Ahmed et al., 2023), whereas others continue to observe B2 as the dominant phylogroup among UTI-associated isolates. Salmani et al. (2016) reported the predominance of phylotype B2 (47.5%), with group B1 accounting for 2.5%. Another study in Iran reported that *E. coli* isolates from urine and stool samples belonged to group B2 (55.2%), followed by groups A (23.4%) and B1 (10.7%) (Norouzian et al., 2019). The high frequency of group B2 among *E. coli* isolates from urine, followed by groups B1 and A, was also observed (Rigvava et al., 2023). Moreover, in Ethiopia, the majority of *E. coli* isolates were phylogroup B2 (60, 30%) followed by D (55, 27.5%), B1 (48, 24%) and A (37, 18.5%) (Dadi et al., 2020). These discrepancies may reflect geographic variations, differences in patient populations, antimicrobial selective pressure, and ecological differences (Norouzian et al., 2019).

The virulence gene profile observed in ESBL-producing *E. coli* isolates in the current study provides insights into the pathogenic potential of these resistant isolates.

The virulence gene profile identified among ESBL-producing isolates suggests that resistance and pathogenicity traits may coexist within the same genetic background. Adhesion-associated determinants such as *fim*H were detected in all the isolates, consistent with their established role in urinary tract colonization and persistence (Bunduki et al., 2021; Golpasand et al., 2024). Similar high frequencies of *fim*H have been reported among UTI-associated isolates in different geographic regions (Dadi et al., 2020; Hasan et al., 2022; Mahshouri et al., 2025).

The presence of the *hly*A gene further indicates the potential for enhanced tissue damage and invasive capacity, although reported prevalences vary considerably among studies (Farshad and Emamghorashi, 2009; Tarchouna et al., 2013; Hasan and Ibrahim, 2022). A study in Egypt reported the detection of *hlyA* gene from 10% of stool isolates and 44% of urine isolates (Osman et al., 2012). The coexistence of such virulence determinants in ESBL-producing *E. coli* enhances their ability to adhere, invade, and colonize the host, thus increasing the pathogenicity of the bacteria (Schroeder et al., 2017; Srivastava et al., 2019).

Shiga toxin type 1 and type 2, the virulence factors responsible for bloody diarrhea and hemolytic–uremic syndrome (HUS), are mainly produced by Shiga toxin-producing *E. coli* (STEC). Toval et al. (2014) hypothesized that Stxs expressed by EHEC strains colonizing the urinary tract may have a virulence-associated function and could injure the human urothelium.

The *stx2* gene is more commonly associated with gastrointestinal infection; however, it can be isolated from ESBL-producing *E. coli* isolates from patients with UTI. Similarly, a study in Iran reported the presence of *stx2* in 6% of *E. coli* isolates from patients with UTI (Nazemi et al., 2012). In Egypt, Osman et al. (2012) reported the high prevalence of the *stx1* gene in 27% of *E. coli* isolates from urine samples; however, the study revealed that *stx2* was not identified.

A similar hypothesis has been proposed regarding the horizontal transfer and emergence of strains carrying both uropathogenic and Shiga toxin-associated determinants (Johnson et al., 2001; Nazemi et al., 2012).

The REP-PCR method is appropriate for epidemiological studies of most bacteria along with pulsed field gel electrophoresis (Pitout et al., 2009). This method has the advantages of applicability, ease of use, high distinction power, and speed (Tafvizi and Tajabadi Ebrahimi, 2015). In the present study, REP-PCR analysis demonstrated substantial genetic diversity among ESBL-producing *E. coli* isolates, thus supporting the heterogeneous nature of strains associated with UTIs. Similar studies have reported considerable clonal diversity among clinical *E. coli* isolates using REP-PCR (Dombek et al., 2000; Kabore et al., 2022).

High genetic variability among uropathogenic strains has also been reported in different geographic settings (Ardakani and Ranjbar, 2016; Movahedi et al., 2021; Mahshouri et al., 2025). This genetic diversity is a challenge for the control and treatment of UTIs caused by ESBL-producing *E. coli* (Mahshouri et al., 2025).

The clustering of certain urine and stool isolates within the same genetic groups suggests the possible epidemiological link between intestinal carriage and urinary infection.

This finding supports the concept that the gastrointestinal tract may serve as a reservoir for UPEC. This hypothesis is supported by Moreno et al. (2008) who reported a relationship between UPEC and the host’s fecal flora. Such diversity, combined with shared clonal patterns, highlights the complexity of transmission dynamics and presents challenges for infection control and therapeutic prevention.

Nanoparticle-based antimicrobial strategies have gained increasing attention as potential alternatives to conventional antibiotics, particularly against MDR pathogens (El-Gazzar and Enan, 2020; Sitohy et al., 2021). In the present study, SeNPs and SeNCs demonstrated notable antibacterial activity against ESBL-producing *E. coli*.

The antimicrobial activity of SeNPs has been attributed to their ability to interact with bacterial cell membranes, disrupt membrane integrity, induce oxidative stress, and interfere with essential cellular processes (Abou Elez et al., 2021).

The information employed herein demonstrated pure area using the DLS method for SeNPs and SeNCs at 70.8 and 99.1 nm, respectively, as demonstrated in the previous study of El-Gazzar and Ismail (2020). TEM investigation revealed that the SeNPs in the solution were distributed evenly without clumping together and came in a range of forms. Given that these compounds function as capping and agglomeration-preventing agents as exhibited in FTIR, our findings may contribute to the explanation of the variety of molecules involved in the formation of SeNPs (El-Gazzar and Ismail, 2020). Peak assignments were made by comparison with published literature values for biogenic SeNPs and CEP vibrational analysis including DFT-validated assignments by Faur et al. (2024). Shifts of 5–20 cm^−1^ in key functional group peaks between individual components and the nanocomposite were interpreted as evidence of molecular interaction. The x-ray diffraction investigation verified the presence of characteristic peaks and a cubic lattice of SeNPs in a novel CEP nanocomposite (El-Gazzar et al., 2025). According to El-Gazzar and Ismail (2020), the solitary peak that was observed indicated that the biosynthesized nanoparticles are of satisfactory quality. SeNPs were dispersed in round and oval shapes. Furthermore, the outcomes established the existence of several molecules responsible for the synthesis and stabilization of SeNPs, which is consistent with earlier findings in this field (El-Gazzar et al., 2025). The observed temporal stability validates the zeta potential measurements and confirms robust colloidal stabilization through electrostatic repulsion mechanisms.

Depending on the concentration and size of the nanoparticles, their inhibitory actions can change (Sitohy et al., 2021). In the current data, SeNCs exhibited significantly higher antibacterial activities than SeNPs or CEP. When compared to SeNPs alone and the traditional antibiotic (CEP), MIC testing showed that SeNCs had much higher antibacterial activity. These results highlight the significance of nanoparticle architecture and surface characteristics in influencing activity and show that the nanocomposite formulation improves antibacterial efficacy beyond that of single-component systems. These findings match with Riyal et al. (2023) who showed that pairing two drugs produces a synergistic interaction that produces an inhibitory impact larger than the total of the effects of the agents alone. A different earlier study found that chitosan and SeNPs worked well together to treat bacteria that were resistant to drugs. According to Alghuthaymi et al. (2021), the kind of microorganism determines the interaction between the concentration of nanoparticles and the antibacterial agent. Also, a previous study found that drug-resistant bacteria might be successfully cured by combining silver nanoparticles with fluconazole or voriconazole. Furthermore, earlier research showed that MnNPs, either alone or in combination with different antibiotics, were effective versus *Pseudomonas aeruginosa, Klebsiella pneumoniae*, and *E. coli* at 18, 14, and 12 mm. Furthermore, TEM in the present study showed that SeNC-treated *E. coli* had significant morphological changes, such as cytoplasmic condensation, membrane rupture, and cell wall deformation. Untreated controls maintained normal morphology and undamaged membranes, demonstrating that exposure to nanoparticles caused these structural alterations. The increased antibacterial effects suggested by MIC data are directly supported by these observations.

The potential activity of SeNPs with CEP is attributed to their large surface area and nanosize. This facilitates the entry, integration, and distribution of CEP materials within the cells, allowing them to more effectively interact with cellular structures and transfer channels (El-Gazzar et al., 2025). According to earlier research, the *E. coli* cell was disrupted by the silver load with MnO_2_ (Wang et al., 2016). The current study indicates that SeNCs have a very simple binding mechanism and can easily pass through microbial structures, such as cell walls leading to alterations in membrane integrity and permeability. Such membrane perturbations may facilitate increased intracellular access of the associated antibiotic, thereby enhancing its antibacterial efficacy. In addition to membrane-associated effects, SeNCs may contribute to antibacterial activities through the induction of oxidative stress. The generation of reactive oxygen species (ROS) can compromise essential cellular components, including membrane lipids, proteins, and nucleic acids, ultimately sensitizing bacterial cells to antibiotic-mediated damage, where the morphological changes shown in TEM micrographs are consistent with enhanced bacterial susceptibility and cellular damage caused by elevated ROS. Thus, one possible explanation for the higher activity of SeNCs is the combination of oxidative stress and membrane disruption (Riyal et al., 2023). Moreover, their ability to stimulate *E. coli* morphogenesis is in line with the results of an earlier investigation (El-Gazzar et al., 2025). Furthermore, by interfering with efflux pump systems, interactions between SeNCs and bacterial membranes may improve the intracellular retention of related antibacterial drugs (Riyal et al., 2023).

Although the clinical isolates showed a variety of resistance genotypes and morphologies, these findings mainly provide context to highlight the originality and applicability of therapies based on nanoparticles. According to the data, SeNCs may be used as antibacterial adjuvants rather than as a substitute for traditional antibiotics as they can successfully target types of bacteria that are resistant to several drugs.

### Limitations

Colistin susceptibility was not evaluated, and standardized broth microdilution testing would be necessary in future studies to assess resistance to this antibiotic. The molecular screening of targeted selected β-lactamase genes did not include *bla*CTX-M, currently the most prevalent ESBL gene family, thereby limiting comprehensive genotypic assessment. In addition, the study relied on *in vitro* antibacterial assays to evaluate the selenium nanomaterials. No cytotoxicity testing on mammalian cell lines or *in vivo* efficacy studies were performed, and therefore, the safety and transitional applicability of these nanomaterials remain to be determined.

## Conclusions

The present study highlights the occurrence of MDR ESBL-producing *E. coli* isolates harboring virulence-associated genes in urine and stool samples. The observed coexistence of resistance and virulence determinants underscores the epidemiological complexity of UTI-associated strains. SeNPs and SeNCs demonstrated *in vitro* antibacterial activities against the examined isolates, with SeNCs exhibiting enhanced inhibitory effects compared to SeNPs and CEP alone. These findings provide preliminary evidence supporting the potential of selenium-based nanomaterials as alternative antimicrobial strategies. Future studies should validate these findings on more recent and large cohorts to better reflect evolving AMR trends. Expanded molecular screening including blaCT-M and other clinically significant resistance genes would provide a more comprehensive genotypic profile. Finally, *in vivo* efficacy and toxicity evaluation in appropriate animal models is essential to assess the safety of selenium-based nanocomposites.

## Data Availability

The datasets presented in this study can be found in online repositories. The names of the repository/repositories and accession number(s) can be found in the article/**Supplementary Material**.
