# Supplementary material for: Molecular characterization of ESBL-producing Escherichia coli from UTIs and the antimicrobial activity of biosynthesized selenium nanocomposites
Source: Front Cell Infect Microbiol. 2026 Apr 7;16:1774867. doi: 10.3389/fcimb.2026.1774867 (PMC13096076; doi:10.3389/fcimb.2026.1774867)
Supplement: Supplementary file 1 [file Supplementaryfile1.docx]

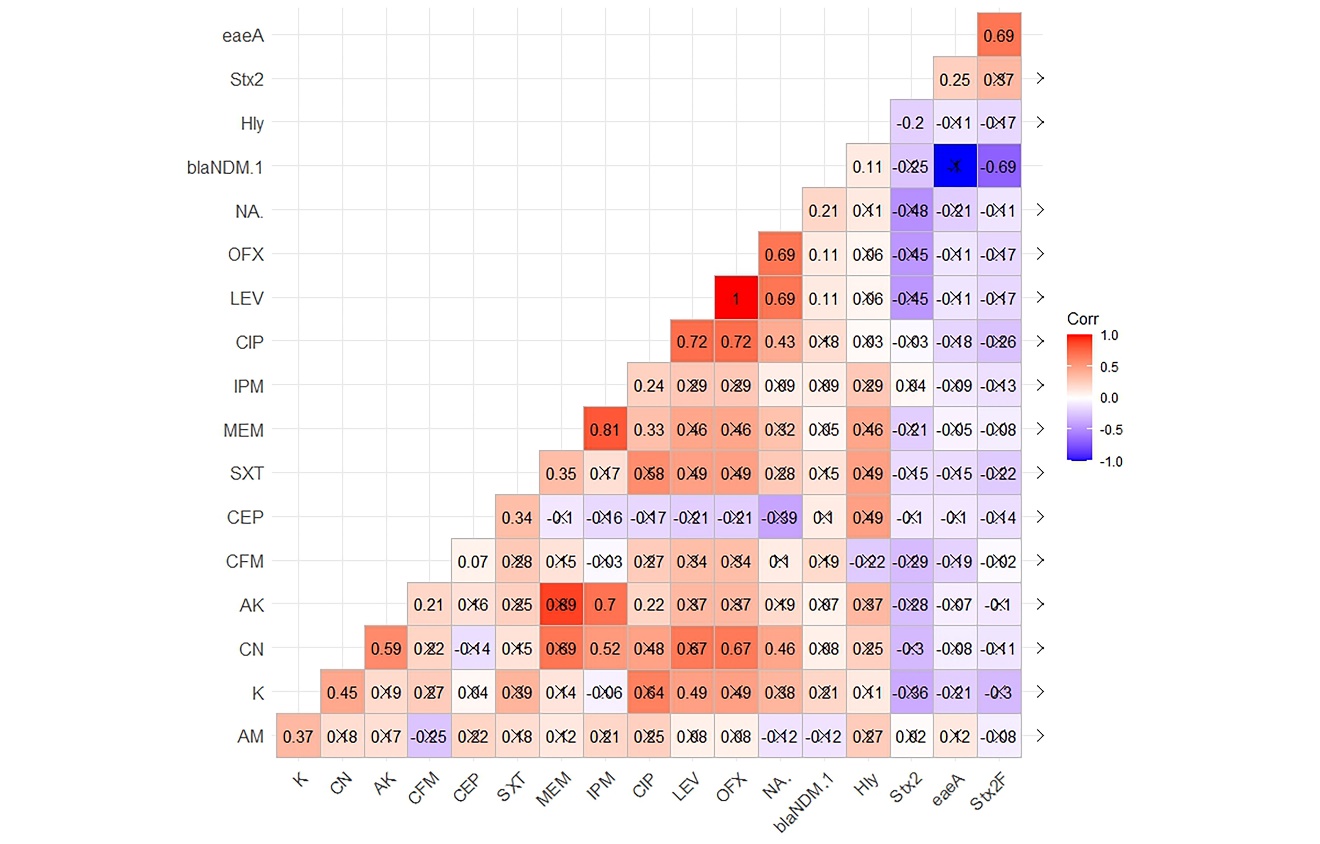


**Supplementary Figure S1. Pairwise correlation (*r*) between phenotypic antimicrobial resistance, β-lactam resistance genes, and virulence genes of ESBL-producing *Escherichia coli* isolates. The scale on the right of the figure refers to the correlation coefficient (*r*). The more intense the color, the more the stronger the positive or negative correlation. Statistical significance was determined using Benjamini–Hochberg false discovery rate (FDR)–adjusted p-values to account for multiple comparisons. X marks indicate insignificant correlations (adjusted *p* > 0.05). Variables that are identical among all strains are excluded and thus not shown in this figure.**

**AM: Ampicillin, K: Kanamycin, CN: Gentamicin, AK: Amikacin, CFM: cefixime, CEP: Cefoperazone, SXT: Trimethoprim/sulfamethoxazole, MEM: Meropenem, IPM: Imipenem, CIP: Ciprofloxacin, LEV: Levofloxacin, OFX: Ofloxacin, NA: Nalidixic acid, *bla*: β-lactamase, *hly*: hemolysin O precursor, *Stx2*: Shiga toxin 2 subunit, *eaeA:* intimin adherence protein.**


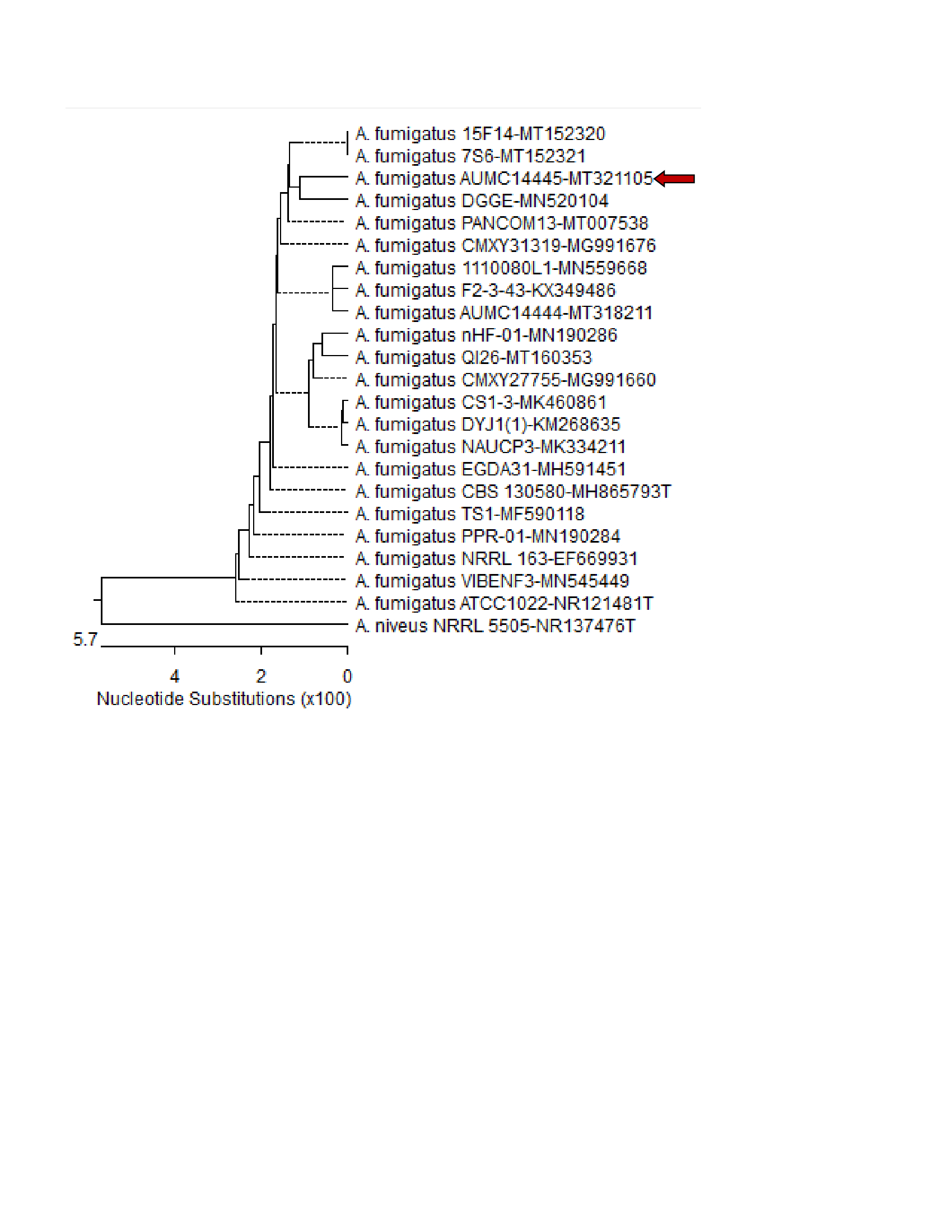


**Supplementary Figure S2.** Phylogenetic tree based on ITS sequences of rDNA of *A. fumigatus* AUMC14445 (arrowed) isolated in the present aligned with closely related sequences accessed from the GenBank. (*A.* = *Aspergillus*). *A. fumigatus* with accession no. [MT321105](https://www.ncbi.nlm.nih.gov/nuccore/MT321104)


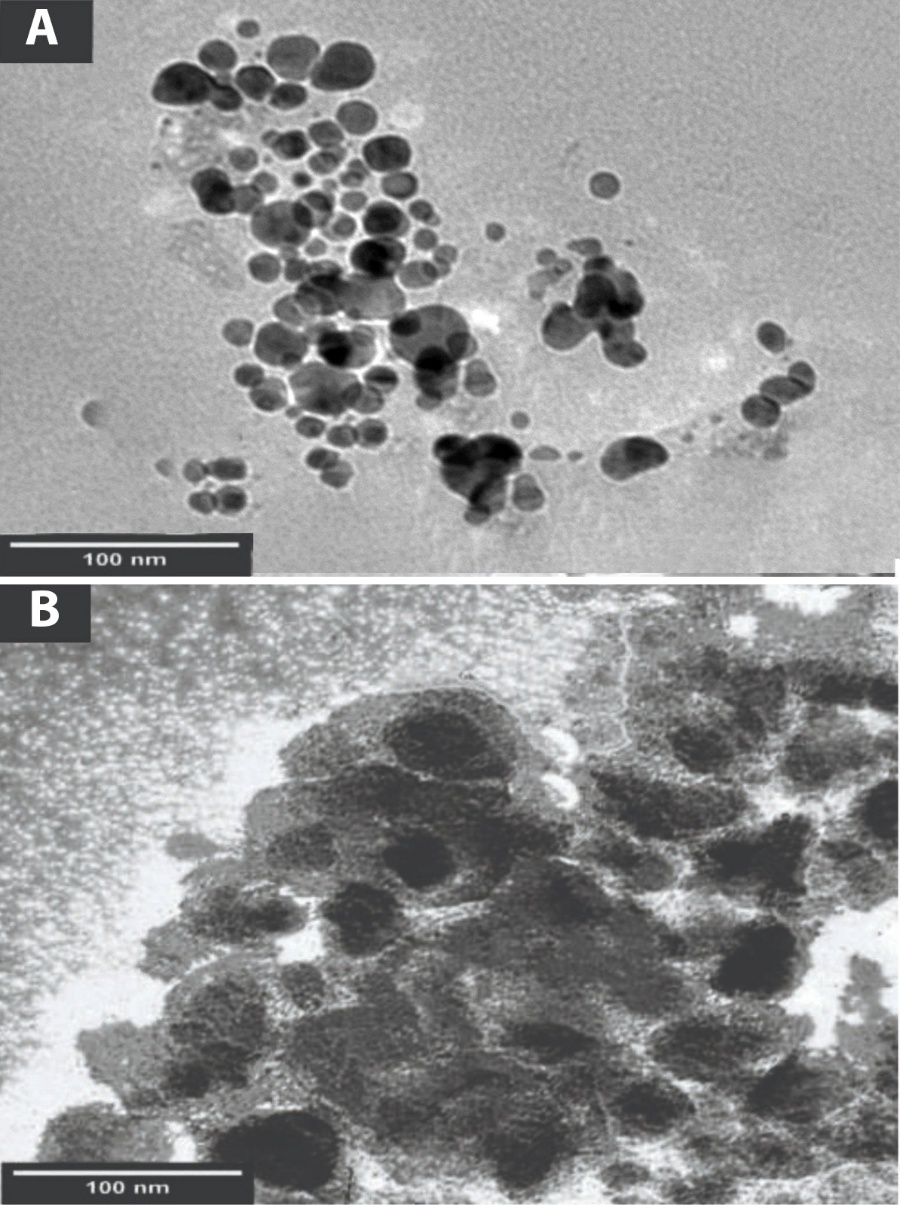


**Supplementary Figure S3.** Transmission electron microscopy of nanoparticles, **(A)**: SeNPs, **(B):** SeNCs
