# Supplementary material for: Molecular characterization of ESBL-producing Escherichia coli from UTIs and the antimicrobial activity of biosynthesized selenium nanocomposites
Source: Front Cell Infect Microbiol. 2026 Apr 7;16:1774867. doi: 10.3389/fcimb.2026.1774867 (PMC13096076; doi:10.3389/fcimb.2026.1774867)
Supplement: Supplementary file 2 [file Table1.docx]

**Supplementary Table (1): Oligonucleotide primers, product length, annealing temperature for PCR reactions used for the amplification of different genes in *Escherichia coli* isolates**.

| **Reference** | **Annealing** | **Length of amplified product** | **Primer sequence**  **(5'-3')** | **Gene** |
| --- | --- | --- | --- | --- |
| **(**[**Hu *et al*., 2011**](#_ENREF_92)**)** | 55˚C  40 sec. | 720 bp | CGATTCTGGAAATGGCAAAAG | ***pho*A** |
|  |  |  | CGTGATCAGCGGTGACTATGAC |  |
| **(**[**Xia *et al*., 2012**](#_ENREF_237)**)** | 53˚C  30 sec. | 280 bp | AGTGGTGAGTATCCGACA | ***bla*VIM** |
|  |  |  | ATGAAAGTGCGTGGAGAC |  |
|  | 53˚C  40 sec. | 488 bp | CATGGTTTGGTGGTTCTTGT | ***bla*IMP** |
|  |  |  | ATAATTTGGCGGACTTTGGC |  |
|  | 55˚C  30 sec. | 287 bp | GGCGGAATGGCTCATCACGA | ***NDM1*** |
|  |  |  | CGCAACACAGCCTGACTTTC |  |
| **(**[**Colom *et al*., 2003**](#_ENREF_30)**)** | 54˚C  40 sec. | 516 bp | ATCAGCAATAAACCAGC | ***bla*TEM** |
|  |  |  | CCCCGAAGAACGTTTTC |  |
|  | 54˚C  40 sec. | 392 bp | AGGATTGACTGCCTTTTTG | ***bla*SHV** |
|  |  |  | ATTTGCTGATTTCGCTCG |  |
| **(**[**Jeong *et al*., 2012**](#_ENREF_102)**)** | 55˚C  30 sec. | 279 bp | GAC GAA CCA ACG GTC AGG AT | ***chu*A** |
|  |  |  | TGC CGC CAG TAC CAA AGA CA |  |
|  | 55˚C  30 sec. | 211 bp | TGA AGT GTC AGG AGA YGC TG | ***yja*A** |
|  |  |  | ATG RAG AAT GCG TTC CTC AAC |  |
|  | 55˚C  30 sec. | 152 bp | GAG TAA TGT CGG GGC ATT CA | ***tsp*E4C2** |
|  |  |  | CGC GYC AAC AAA GTA TTR CG |  |
| **(**[**Ghanbarpour and Salehi, 2010**](#_ENREF_76)**)** | 50˚C  40 sec. | 508 bp | TGCAGAACGGATAAGCCGTGG | ***fim*H** |
|  |  |  | GCAGTCACCTGCCCTCCGGTA |  |
| **(**[**Piva *et al*., 2003**](#_ENREF_173)**)** | 60˚C  40 sec. | 1177 bp | AACAAGGATAAGCACTGTTCTGGCT | ***Hly*** |
|  |  |  | ACCATATAAGCGGTCATTCCCGTCA |  |
| **(**[**Dipineto *et al*., 2006**](#_ENREF_41)**)** | 58˚C  40 sec. | 779 bp | CCATGACAACGGACAGCAGTT | ***Stx*2** |
|  |  |  | CCTGTCAACTGAGCAGCACTTTG |  |
| **(**[**Bisi-Johnson *et al*., 2011**](#_ENREF_21)**)** | 51˚C  30 sec. | 248 bp | ATGCTTAGTGCTGGTTTAGG | ***eae*A** |
|  |  |  | GCCTTCATCATTTCGCTTTC |  |
| **(**[**Schmidt *et al*., 2000**](#_ENREF_197)**)** | 57˚C  40 sec. | 428 bp | AGA TTG GGC GTC ATT CAC TGG TTG | ***Stx*2F** |
|  |  |  | TAC TTT AAT GGC CGC CCT GTC TCC |  |
| **(**[**Mohapatra *et al*., 2007**](#_ENREF_148)**)** | 40˚C  1 min. | Variable | IIIICGICGICATCIGGC | **REP** |
|  |  |  | ICGICTTATCIGGCCTAC |  |

bp: base pair, *phoA* : alkaline phosphatase **,** *bla*: B-lactamase, *VIM*: Verona integron–encoded metallo-B-lactamase, IMP: imipenem, *NDM*: New Delhi Metallao Betalactamase, *TEM*: Temoneria (the name of the patient from which the isolate was recovered), *SHV*: sulfyhdryl variable, *chuA: heme binding protein, yjaA: stress response protein, tspE4C2:* *anonymous DNA fragment,* *fimH :* type 1 fimbriae D-mannose specific adhesion , *hly* : hemolysin O precursor , *Stx2* : Shiga toxin 2 subunit , *eaeA : intimin adherence protein*, *REP* : Repetitive Extragenic Palindromic
